# SAPCD2 promotes neuroblastoma progression by altering the subcellular distribution of E2F7

**DOI:** 10.1038/s41419-022-04624-z

**Published:** 2022-02-23

**Authors:** Zi-Mu Zhang, Hai-Bo Cao, Zhi-Heng Li, Ran Zhuo, Yan-Fang Tao, Xiao-Lu Li, Gen Li, Xin-Mei Liao, Fang Fang, Yi Xie, Di Wu, Hai-Rong Wang, Jian-Wei Wang, Yan-Ling Chen, Juan-Juan Yu, Si-Qi Jia, Ran-Dong Yang, Xin-Yi Guo, Yang Yang, Chen-Xi Feng, Yun-Yun Xu, Guang-Hui Qian, Jian Pan

**Affiliations:** 1 Institute of Pediatric Research, Children’s Hospital of Soochow University, Suzhou, 215003 Jiangsu China; 2 Department of Pediatrics, The Affiliated Hospital of Yangzhou University, Yangzhou, 225000 Jiangsu China; 3 School of Electronic Information and Electrical Engineering, Shanghai Jiao Tong University, Shanghai, 200000 China; 4 School of Basic Medicine and Biological Sciences, Soochow University, Suzhou, 215123 Jiangsu China; 5 Medical College of Soochow University, Suzhou, 215123 Jiangsu China

**Keywords:** Cancer, Cancer

## Abstract

Recent studies uncovered the emerging roles of SAPCD2 (suppressor anaphase-promoting complex domain containing 2) in several types of human cancer. However, the functions and underlying mechanisms of SAPCD2 in the progression of neuroblastoma (NB) remain elusive. Herein, through integrative analysis of public datasets and regulatory network of GSK-J4, a small-molecule drug with anti-NB activity, we identified SAPCD2 as an appealing target with a high connection to poor prognosis in NB. SAPCD2 promoted NB progression in vitro and in vivo. Mechanistically, SAPCD2 could directly bind to cytoplasmic E2F7 but not E2F1, alter the subcellular distribution of E2F7 and regulate E2F activity. Among the E2F family members, the roles of E2F7 in NB are poorly understood. We found that an increasing level of nuclear E2F7 was induced by SAPCD2 knockdown, thereby affecting the expression of genes involved in the cell cycle and chromosome instability. In addition, Selinexor (KTP-330), a clinically available inhibitor of exportin 1 (XPO1), could induce nuclear accumulation of E2F7 and suppress the growth of NB. Overall, our studies suggested a previously unrecognized role of SAPCD2 in the E2F signaling pathway and a potential therapeutic approach for NB, as well as clues for understanding the differences in subcellular distribution of E2F1 and E2F7 during their nucleocytoplasmic shuttling.

## Introduction

Neuroblastoma (NB) is one of the most common malignant extracranial solid tumors in pediatric patients that originates from neural crest cells of the sympathetic nervous system and accounts for up to 15% of cancer-related mortality in children [1]. Rather than the gradual accumulation of oncogenic driver events led by hereditary and environmental factors as well as replication errors in adult tumors, pediatric tumors are mostly driven by mutations, variants in key genes or abnormal activation of key pathways within certain critical stem/progenitor cells at specific periods of development, leading to the initiation and progression of tumors [2, 3]. Although several genomic and genetic aberrations, such as MYCN amplification, ALK mutation, PHOX2B mutation, and single nucleotide polymorphisms within LIN28B, have been found to be significantly associated with a poor outcome [4], further investigations on mechanisms driving NB progression are still warranted to provide insights for improving the prognosis of patients.

GSK-J4 is a novel small-molecule inhibitor of lysine 27 of histone 3 (H3K27) demethylases. In a recent study, of about 800 solid tumor cancer cell lines tested, NB cell lines displayed the most reduced cell viability after GSK-J4 treatment [5]. The efficacy of GSK-J4 monotherapy in NB has been confirmed by in vitro and in vivo studies in animals, and JMJD3 (histone demethylase Jumonji D3, also called KDM6B) was identified as a key target of GSK-J4 in NB [5, 6]. With the aim to discover new players involved in the progression of NB, we performed bioinformatics analysis of the GSK-J4 regulatory network and identified 19 genes that are significantly downregulated in multiple GSK-J4-treated NB cell lines. Among those genes, SAPCD2 (suppressor anaphase-promoting complex domain containing 2) emerged as an appealing target for further investigations given its high connection to poor patient prognosis and in consideration of the lack of understanding of its roles and regulatory mechanisms in NB.

SAPCD2, also known as C9orf140 or p42.3, is located in the 9q34.3 site of the human chromosome and encodes a 389-amino acid protein with EF-Hand domain at the N-terminal and functional coil–coil(CC)-domains at the C-terminus [7]. SAPCD2, initially identified as a cell cycle-dependent gene in gastric cancer, has been found to preferentially express in the M and G1 phases over the S and G2 phases, and act as a key regulator of controlling spindle orientation and divisions [8, 9]. Accumulating studies have shown an elevated expression level of SAPCD2 in a number of human cancers, such as colorectal cancer, fibrosarcoma, melanoma, and non-small cell lung cancer. SAPCD2 largely functions as an oncogene playing important roles in tumorigenesis by promoting cell proliferation, cell migration, and invasion by various pathways, such as PI3K/Akt activation, MAPK activation, or Hippo signaling suppression [10–13]. A recent study, however, suggested that SAPCD2 blocks Wnt/β-catenin signaling by interaction with Axin1 and in some contexts might function as a tumor suppressor [14].

In our study, functional analyses in cultured NB cells and xenograft mice models demonstrated that SAPCD2 played critical roles in sustaining NB cells growth and survival. Transcriptome analysis revealed that SAPCD2-knockdown limited E2F signaling activation in NB cells affected the expression of genes involved in the cell cycle and chromosome instability. Mechanistical studies showed that through direct binding to cytoplasmic E2F7, one of the transcriptional repressors in the E2F family, SAPCD2 altered the subcellular distribution of E2F7 and decreased the level of nuclear E2F7 in NB cells, thus restraining the inhibitory effect of nuclear E2F7 on E2F signaling pathway. Furthermore, we suggested that Selinexor (KTP-330), a clinically available inhibitor of exportin 1 (XPO1) protein, could suppress the growth of NB by inducing nuclear accumulation of E2F7, and combine with GSK-J4 to enhance NB regression. Overall, our study provided evidences of a previously unrecognized role of SAPCD2 in the E2F signaling pathway and revealed a novel target and potential therapeutic strategy for clinical NB treatment.

## Results

### SAPCD2 is associated with the NB progression

**Fig. 1:**
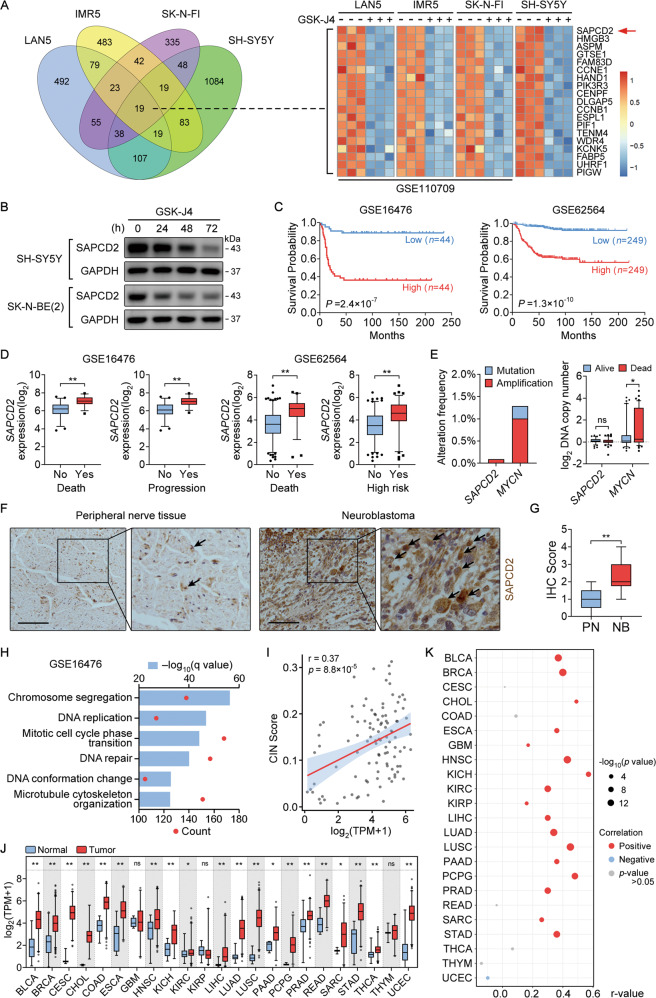
Identification of SAPCD2 as a prognostic factor associated with NB progression. **A** Venn diagram and heatmap revealing the identification of downregulated genes in GSK-J4-treated NB cell lines using the public dataset (GSE110709) and our previous microarray study (GSE180601). **B** Western blot indicating the expression of SAPCD2 in SH-SY5Y treated with 0.5 μM GSK-J4 and SK-N-BE(2) treated with 1 μM GSK-J4 for time points as indicated. **C**. Kaplan–Meier curves indicate the survival of NB patients with high or low SAPCD2 expression. **D** Public datasets reveal the differential expression of SAPCD2 transcript in NB tissues with different status of death, progression, or risk of progression. **E** Public database revealing the copy number and genetic variants of *MYCN* and *SAPCD2* gene in NB tissues with different status of death. **F**, **G** Representative immunohistochemical staining and immunohistochemistry scores revealing SAPCD2 expression in NB tissues and normal peripheral nerve tissues (PT). Scale bars, 100 μm. **H** GO analysis of the top 50% of genes (*n* = 2311 genes) that tightly co-expressed with SAPCD2. FDR <0.05. **I** Scatter plot shows a positive significant correlation between SAPCD2 transcript levels and CIN scores in NB tissues. **J** SAPCD2 expression levels relative to the indicated tumor types and normal samples from the TCGA dataset. **K** Bubble plot showing levels of correlation between SAPCD2 transcript levels and CIN scores in indicated tumor types from the TCGA dataset. Log-rank test for analysis in **C**; unpaired two-sided *t*-test in **D**, **E**, **G**, and **J**, data were shown as mean ± SD (error bars); Pearson’s correlation coefficient in **I** and **K**. ns, not significant. Data were representative of three independent experiments in **B**.

GSK-J4 is a small-molecule inhibitor of lysine 27 of histone 3 (H3K27) demethylases and has been shown to have anti-NB efficacy in a range of NB cell lines. To examine the genes essential for NB progression, we analyzed the publicly available RNA-seq datasets of NB cell lines (IMR5, LAN5, and SK-N-FI) treated with GSK-J4 (GSE110709) derived from Gene Expression Omnibus (GEO) and our previous microarray study of GSK-J4-treated NB cell line SH-SY5Y (Fig. 1A). Compared to the control group, we identified 19 downregulated genes shared by distinct NB cell lines treated with GSK-J4. Among these genes, western blot analysis revealed that SAPCD2 levels were reduced in GSK-J4-treated SH-SY5Y and SK-N-BE(2) (Fig. 1B), and 88 (GSE16476) and 498 (GSE62564) NB cases revealed that a high level of SAPCD2 was a strong predictor of a poor outcome (Fig. 1C). In addition, compared to the control group, SAPCD2 was overexpressed in NB tissues with death, progression, or high risk (Fig. 1D). Sequencing data derived from the cBioPortal database showed that alterations in the SAPCD2 gene were infrequent events in NB while the well-established key proto‐oncogene MYCN [4, 15] displayed a high frequency of amplification. The copy number of the SAPCD2 gene was not significantly associated with the death of NB cases derived from the Oncogenomics database (Fig. 1E). Immunohistochemistry staining observed that SAPCD2 protein was strongly expressed in NB tissues but relatively weakly in normal peripheral nerve tissue (Fig. 1F, G). SAPCD2 was also found to be significantly highly expressed in NB in comparison to ganglioneuroma (GN), the benign representative of peripheral neuroblastic tumors, by analyzing a recent study [16] which performed RNA-Seq on GN and NB samples (Supplementary Fig. S1).

To investigate the potential functions of SAPCD2, we then conducted Gene Ontology (GO) enrichment analysis on SAPCD2-correlated genes in 88 specimens (GSE16476), and found that GO terms were significantly enriched in biological processes implicated in chromosome stability, such as chromosome segregation, DNA conformation change and microtubule cytoskeleton organization (Fig. 1H), which play an important role in tumor development and aggressiveness [17]. To further explore the association of SAPCD2 with chromosome stability, we analyzed gene expression and copy number alteration (CNA) data of NB patients from the Therapeutically Applicable Research to Generate Effective Treatments (TARGET) database. The correlation analysis showed a significant positive link between SAPCD2 transcript levels and chromosome instability index (CIN) scores which were defined in a previous study [18] and calculated from CNAs in tumor samples to evaluate the degree of CIN (Fig. 1I). Furthermore, the analysis of diverse datasets from The Cancer Genome Atlas (TCGA) indicated higher levels of SAPCD2 across a number of tumors in comparison to normal samples (Fig. 1J), as well as a positive correlation between SAPCD2 transcript levels and CIN in multiple tumors (Fig. 1K and Supplementary Fig. S2). Collectively, these data revealed that SAPCD2 might have clinical implications in NB and perhaps other tumor types.

### SAPCD2 sustains NB cells growth and survival

**Fig. 2:**
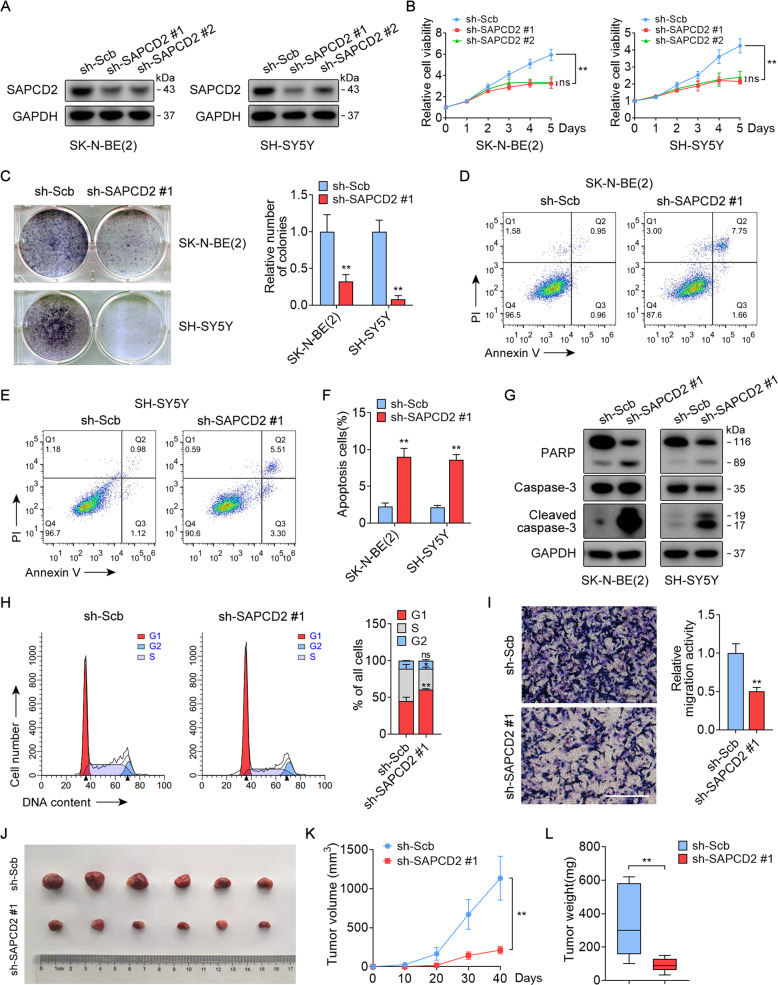
SAPCD2 sustains NB cells growth and survival in vitro and in vivo. **A** Western blot revealing SAPCD2 expression in NB cells stably transfected with scrambled shRNA (sh-Scb) or sh-SAPCD2. **B** CCK-8 assay indicates the change in cell viability of NB cells transfected with sh-Scb or sh-SAPCD2. **C** Representative images and quantification of colony formation assay depicting the growth of NB cells transfected with sh-Scb or sh-SAPCD2. **D**–**F** Flow cytometry showing the apoptosis of NB cells transfected with sh-Scb or sh-SAPCD2. **G** Western blot revealing caspase-3 activation and cleavage of PARP in NB cells transfected with sh-Scb or sh-SAPCD2. **H** Flow cytometry showing cell cycle distribution of SK-N-BE(2) cells transfected with sh-Scb or sh-SAPCD2. **I** Representative images and quantification of transwell assay showing the cell migration of SK-N-BE(2) cells transfected with sh-Scb or sh-SAPCD2. Scale bars, 200 μm. **J**–**L** Representative images, in vivo growth curves and tumor weight at the endpoints of subcutaneous xenografts of SK-N-BE(2) cells transfected with sh-Scb or sh-SAPCD2 into the dorsal flanks of BALB/c nude mice (*n* = 6 per group). One-way ANOVA with Bonferroni’s multiple comparison test for analysis in **B** and **K**; unpaired two-sided *t*-test in **C**, **F**, **H**, **I**, and **L**, data were shown as mean ± SD (error bars). ns, not significant. Data were representative of three independent experiments.

We then investigated the functional requirement for SAPCD2 in human NB cell lines. In the SK-N-BE(2) and SH-SY5Y cell lines, SAPCD2-knockdown by two different shRNAs resulted in a significant decrease in SAPCD2 expression (Fig. 2A) and reduced cell proliferation compared to those transfected with scrambled shRNA (Fig. 2B). Colony formation (Fig. 2C) and flow cytometry for apoptosis analysis (Fig. 2D–F) revealed that SAPCD2-knockdown could impair the growth and promote the apoptosis of tumor cells. Consistently, enhanced caspase-3 activation and cleavage of PARP, which are markers of cell apoptosis, were detected in the SAPCD2-knockdown group (Fig. 2G). Cell cycle analysis indicated that SAPCD2-knockdown in SK-N-BE(2) cells led to a substantial increase in cells in the G1 phase, accompanied by a decrease in cells in the S phase (Fig. 2H). In addition, the transwell assay showed that SAPCD2-knockdown inhibited the cell migration efficiency (Fig. 2I). To further investigate the impact of SAPCD2-knockdown in vivo, we established subcutaneous xenografts of SK-N-BE(2) cells, stably expressing an shRNA targeting SAPCD2 or a scramble control in nude mice. SAPCD2-knockdown resulted in a significant reduction in tumor size and weight (Fig. 2J–L). Taken together, our findings suggested that SAPCD2-knockdown impaired NB cell growth and survival in vitro and in vivo.

### Transcriptome analyses show dysregulated E2F activity in SAPCD2-knockdown NB cell

**Fig. 3:**
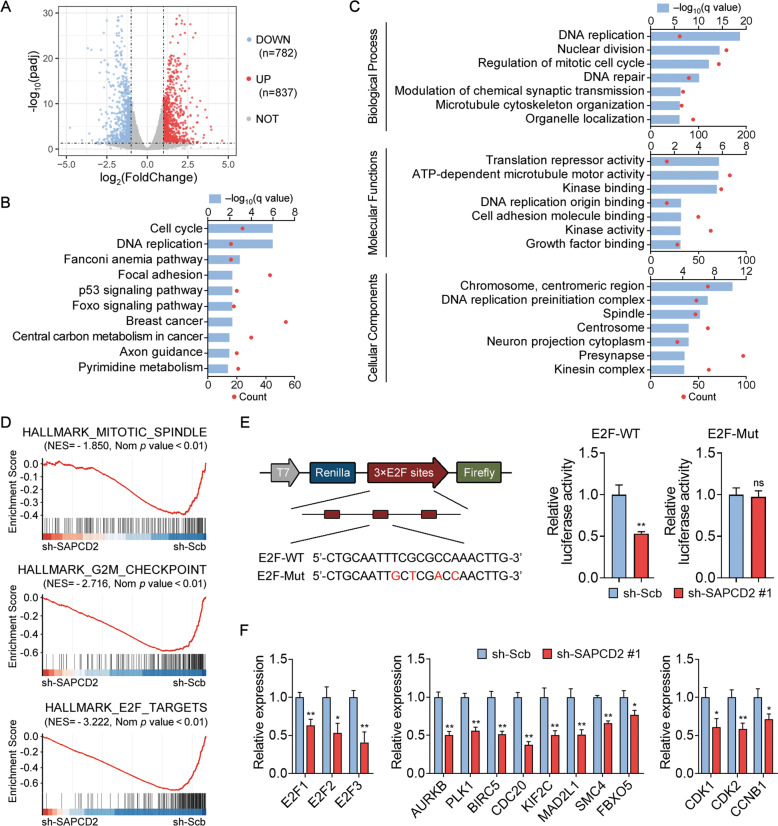
Transcriptome analysis of SAPCD2-dependent regulation. **A** Volcano plot of all expressed genes is shown to identify DEGs obtained from RNA-seq data in SAPCD2-knockdown SK-N-BE(2) cells with abs(log_2_(fold change)) >1 and adjusted *p* value <0.05. **B**, **C** KEGG pathway analysis (**B**) and GO analysis (**C**) for DEGs. **D** Enrichment plots generated from GSEA hallmark gene set using DEGs upon SAPCD2-knockdown. NES normalized enrichment score. NOM *p* value, adjusted *p* value. **E** Activity of the dual-luciferase reporter vector containing WT or mutant E2F-binding motifs that were transfected into SK-N-BE(2) cells transfected with sh-Scb or sh-SAPCD2. **F** qPCR analysis of mRNA levels for the indicated E2F transcription activators, E2F target genes, and cell cycle-related genes in SK-N-BE(2) cells transfected with sh-Scb or sh-SAPCD2. Unpaired two-sided *t*-test for analysis in **E**–**F**, data were shown as mean ± SD (error bars). ns, not significant. Data were representative of three independent experiments.

To explore SAPCD2-dependent gene regulation, RNA-seq analysis was conducted and revealed that 837 genes were upregulated whereas 782 genes were downregulated in the SAPCD2-knockdown SK-N-BE(2) cells in comparison to the control group (Fig. 3A). Kyoto Encyclopedia of Genes and Genomes (KEGG) analysis showed that differentially expressed genes (DEGs) were enriched in the cell cycle, DNA replication, p53 signaling pathways, and several other tumor-associated pathways (Fig. 3B). Gene Ontology (GO) analysis indicated that the cell components most enriched in the DEGs were related to chromosome, DNA replication preinitiation complex, and spindle. These genes were classified into GO terms involved in biological processes such as DNA replication, nuclear division, and regulation of the mitotic cell cycle (Fig. 3C). In addition, Gene Set Enrichment Analysis (GSEA) revealed that the downregulated genes in SAPCD2-knockdown cells were significantly enriched in SAPCD2-positively correlated genes in 88 (GSE16476) and 498 specimens (GSE62564), and vice versa (Supplementary Fig. S3).

GSEA with Hallmark signatures for SAPCD2-knockdown and control group showed significant enrichment for mitotic spindle, G2M checkpoint, and E2F target genes (Fig. 3D and Supplementary Table S1). Given the well-recognized roles of the family of E2F transcription factors in the regulation of cell cycle progression and in chromosome stability, we further assessed the effect of SAPCD2-knockdown on the levels of E2F-mediated transcriptional activity (Fig. 3E). E2F-WT luciferase reporter which has E2F-binding consensus motifs upstream a luciferase reporter gene was widely used to evaluate E2F-mediated transcriptional activation, while E2F-Mut luciferase reporter which contains mutant motifs was used as a negative control [19]. Luciferase reporter assay revealed that knockdown of SAPCD2 led to a decreased luciferase activity in SK-N-BE(2) cells carrying wild-type E2F motifs, but not in cells carrying mutant E2F motifs. Consistently, the qPCR analysis showed that the expression levels of E2F transcription activators E2F1, E2F2, and E2F3 were reduced, and levels of some of the well-established E2F targets were decreased. Furthermore, downregulation of cell cycle-related genes CDK1, CDK2, and CCNB1 was identified in SAPCD2-knockdown cells by qPCR analysis (Fig. 3F).

### SAPCD2 alters the subcellular distribution of E2F7 in NB cells by directly binding to cytoplasmic E2F7

**Fig. 4:**
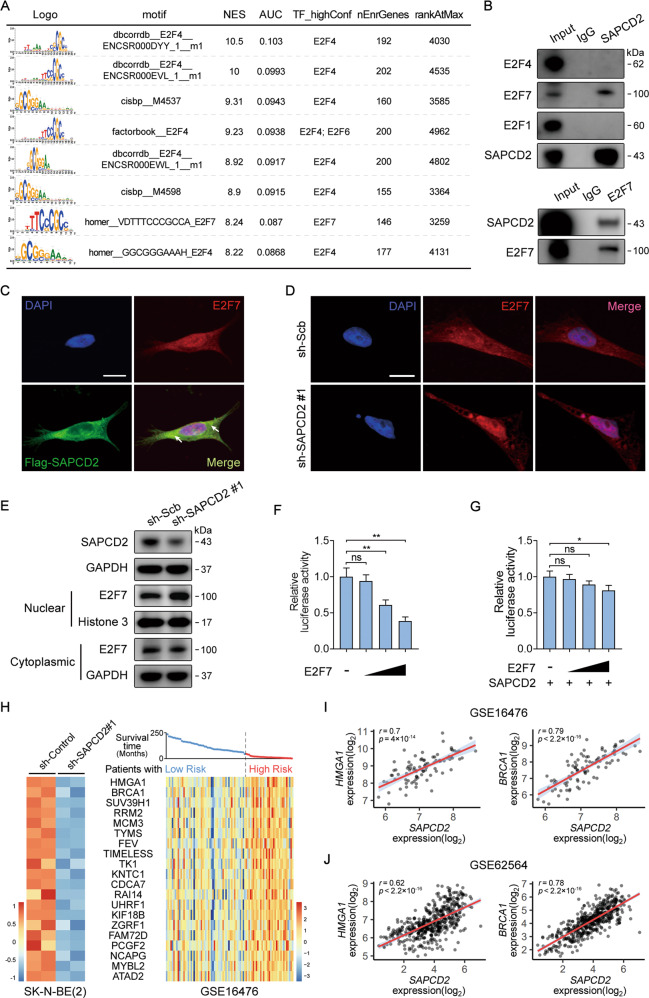
SAPCD2 alters the subcellular distribution of E2F7 in NB cells. **A** Motif enrichment analysis by cisTarget database revealing the potential upstream transcription factors of the downregulated genes in the SAPCD2-knockdown SK-N-BE(2) cells. NES, normalized enrichment score of the motif in the gene set. AUC, area under the curve. TF_highConf, transcription factors annotated to the motif according to the “motifAnnot_highConfCat” dataset. nEnrGenes, Number of genes highly ranked. rankAtMax, Ranking at the maximum enrichment. **B** Immunoprecipitation of endogenous SAPCD2 or E2F7 in SK-N-BE(2) cells analysed by western blot showing an interaction between SAPCD2 and E2F7. Rabbit IgG was used as a negative control. **C** Immunofluorescence confocal images showing the interaction between SAPCD2 and E2F7 in SK-N-BE(2) cells stably transfected with FLAG-tagged SAPCD2. Arrow, SAPCD2 appears to be mainly colocalized with E2F7 in the cytoplasm. Scale bars, 10 μm. **D**, **E** Immunofluorescence confocal images and western blot showing the subcellular distribution of E2F7 in SK-N-BE(2) cells transfected with sh-Scb or sh-SAPCD2 for 72 h. Scale bars, 10 μm. **F**, **G** Activity of E2F luciferase reporter that were co-transfected into 293T cells with increasing amounts of E2F7 expression plasmid (100–500 ng) in the presence or absence of constant amounts (500 ng) of E2F7 expression plasmid. **H** Heatmap revealing the expression of predicted E2F7-targeted genes obtained from RNA-seq data in SAPCD2-knockdown SK-N-BE(2) cells and public dataset of NB patients with different survival time (GSE16476). **I**, **J** Scatter plot showing a positive significant correlation in transcript level between SAPCD2 and HMGA1 or BRCA1 in NB tissues. Unpaired two-sided *t*-test for analysis in **F**, **G**, data were shown as mean ± SD (error bars). ns, not significant. Pearson’s correlation coefficient in **I**, **J**. Data were representative of three independent experiments.

As a further step towards the understanding of the oncogenic mechanisms of SAPCD2, we performed motif enrichment analysis by cisTarget database to identify upstream transcription factors that might target the DEGs in the SAPCD2-knockdown SK-N-BE(2) cells (Supplementary Table S2). Interestingly, E2F4 and several other members of the E2F transcription factors family were significantly enriched and predicted to be potential upstream transcription factors of the downregulated genes (Fig. 4A). The same approach was afterward taken in analyzing the genes enriched in GSEA set HALLMARK_E2F_TARGETS, and E2F7 was the top predicted upstream transcription factors (Supplementary Tables S2). To investigate whether SAPCD2 could interact with the E2F transcription factor to regulate gene expression, co-immunoprecipitation experiments were carried out to examine the interaction between SAPCD2 and E2F4, E2F7 or the key E2F transcription activator E2F1 (Fig. 4B). The results showed that E2F7, but not E2F4 or E2F1, was able to bind to SAPCD2 protein in the lysates of SK-N-BE(2) cells. Immunofluorescence staining (IF) followed by confocal microscopic analysis revealed that SAPCD2 appeared to be mainly colocalized with E2F7 in the cytoplasm of SK-N-BE(2) cells (Fig. 4C).

Previous studies indicated that E2F7 is frequently mislocalized to the cytoplasm, representing actionable pathology in head and neck squamous cell carcinomas (HNSCCs) [20]. Interestingly, prominent cytoplasmic staining of E2F7 was also observed in SK-N-BE(2) cells (Fig. 4C). We thus further investigated whether SAPCD2 affected the subcellular distribution of E2F7. IF analysis revealed that SAPCD2-knockdown led to markedly enhanced nuclear staining and relatively weak cytoplasmic staining of E2F7 (Fig. 4D). Western blot analysis showed that levels of nuclear E2F7 protein were elevated in SAPCD2-knockdown SK-N-BE(2) cells in comparison to the control group (Fig. 4E). Rescue experiments showed that overexpression of SAPCD2 interrupted the translocation of E2F7 into the nucleus (Supplementary Fig. S4). In addition, 293T cells were transfected with increasing amounts of E2F7 or SAPCD2 together with a luciferase reporter plasmid carrying wild-type E2F motifs. Transfection of the E2F7 expression plasmid, consistent with previous studies, led to a significantly decreased E2F transcriptional activity (Fig. 4F). In line with the role of SAPCD2 in contributing to E2F7 mislocalization to the cytoplasm, the suppressive effect of E2F7 on E2F transcriptional activity was counteracted by SAPCD2 overexpression (Fig. 4G).

Transcriptome analysis identified that a number of E2F7-targeted genes were downregulated in SAPCD2-knockdown SK-N-BE(2) cells. A public dataset of 88 NB cases (GSE16476) revealed that these E2F7-targeted genes were overexpressed in patients with high risk in comparison to the low-risk group (Fig. 4H). Among these E2F7-targeted genes, HMGA1 and BRCA1 were representatives playing critical roles in NB [21, 22], whose expression were positively correlated with that of SAPCD2 in NB patients (Fig. 4I, J). The decrease in nuclear E2F7 derepresses E2F signaling transactivation that has important implications in CIN and tumorigenesis. Several E2F-targeted genes associated with CIN [23–25] were downregulated in SAPCD2-knockdown SK-N-BE(2) cells and predicted poor prognosis in NB patients (Supplementary Fig. S5A, B). In addition, the analysis of diverse public datasets indicated a significant positive correlation between SAPCD2 transcript levels and these E2F-targeted genes across multiple tumors (Supplementary Fig. S5C, D).

Together, these data suggested that SAPCD2 contributed to E2F7 mislocalization to the cytoplasm accompanied by a defect in nuclear E2F7-mediated transcriptional repression, and then led to excessive E2F activity.

### Selinexor suppresses the growth of NB and combines with GSK-J4 to enhance NB regression

**Fig. 5:**
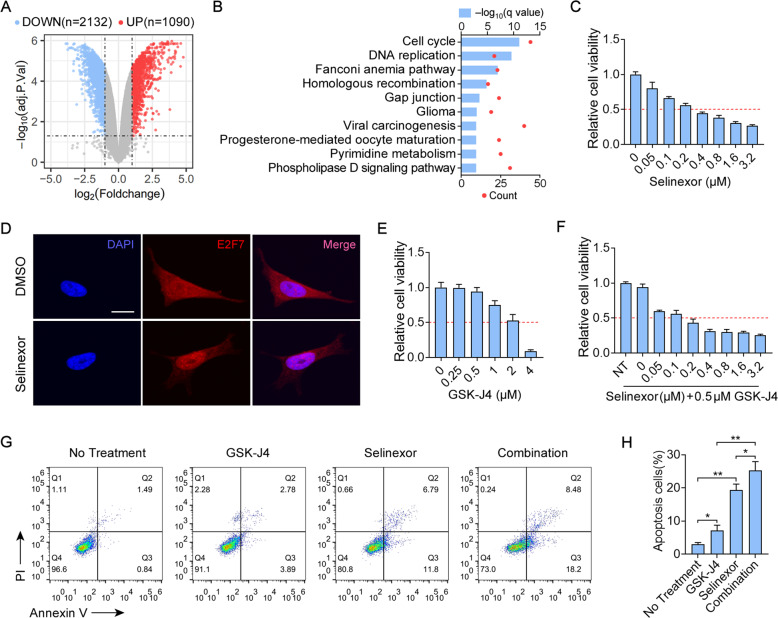
Selinexor suppresses the growth of NB and combines with GSK-J4 to enhance NB regression. **A** Volcano plot of all expressed genes is shown to identify DEGs obtained from microarray study (GSE180601) in Selinexor-treated SK-N-BE(2) cells with abs(log_2_(fold change)) >1 and adjusted *p* value <0.05. **B** KEGG pathway analysis for DEGs. **C** CCK-8 assays for SK-N-BE(2) cells treated with the indicated concentrations of Selinexor for 72 h. **D** Immunofluorescence confocal images showing the subcellular distribution of E2F7 in SK-N-BE(2) cells treated with 0.4 μM Selinexor for 24 h. **E** CCK-8 assays for SK-N-BE(2) cells treated with the indicated concentrations of GSK-J4 for 72 h. **F** CCK-8 assays for SK-N-BE(2) cells treated with 0 or 0.5 μM GSK-J4 and the indicated concentrations of Selinexor for 72 h. **G**, **H** Flow cytometry showing the apoptosis of SK-N-BE(2) cells treated with 0.5 μM GSK-J4, 0.2 μM Selinexor, or the combination for 48 h. Unpaired two-sided *t*-test for analysis in **H**, data were shown as mean ± SD (error bars). Data were representative of three independent experiments.

Selinexor have been previously reported to affect the subcellular distribution of E2F7 and reverse anthracycline resistance in HNSCC [20]. In our previous microarray study (GSE180601), NB cell line SH-SY5Y was treated by Selinexor followed by microarray analysis (Fig. 5A). Interestingly, similar to the KEGG pathways enriched in DEGs of SAPCD2-knockdown, KEGG analysis on DEGs of Selinexor-treated SH-SY5Y cells identified signatures for cell cycle and DNA replication (Fig. 5B). Moreover, Selinexor induced nuclear accumulation of E2F7 and suppressed the growth of SK-N-BE(2) cells (Fig. 5C, D).

Given that SK-N-BE(2) cells have been previously reported to be GSK-J4-resistant cell line [5] and not sensitive to low dose (under 1 μM) of GSK-J4 (Fig. 5E), we asked whether the nuclear accumulation of E2F7 inducted by Selinexor might sensitize SK-N-BE(2) cells to a low dose of GSK-J4. The results showed that the GSK-J4/Selinexor combination significantly suppressed the growth of SK-N-BE(2) cells (Fig. 5F). In addition, an increase in the induction of apoptosis was observed in the combination compared to GSK-J4 or Selinexor single-agent therapy (Fig. 5G).

## Discussion

E2F family members are classified as activators (E2F1, E2F2, and E2F3A), canonical repressors (E2F3B, E2F4, E2F5, and E2F6), and atypical repressors (E2F7 and E2F8) depending on their transcriptional activity and structural features [26]. E2F family members are crucial mediators of cell division and cell fate decisions. E2F switching threshold model was developed in a recent study, which suggested that E2F activity is gradually enhanced during G1 until it reaches a critical level and then allows cell cycle progression to the S phase [27]. This indicates that oncogenic activation of E2F signaling may push the cells from quiescence to unexpected proliferation. In addition, many E2F targets have well-established roles in DNA replication origin licensing and mitosis [28]. Aberrant expression of these E2F targets is tightly associated with chromosome instability (CIN), which is inherent to most human cancers, and may lead to tumors with poor prognosis and drug resistance by expediting the accumulation of advantageous genotypes [29].

Here we presented evidence that SAPCD2 (suppressor anaphase-promoting complex domain containing 2), which we showed as being suppressed in GSK-J4-treated neuroblastoma (NB) cells, was overexpressed in human neuroblastoma (NB) samples and was associated with NB progression and poor outcome of patients. We also discovered the significant correlation of SAPCD2 transcript levels with the degrees of CIN in NB as well as some other tumor types. In addition, our findings indicated that SAPCD2 exerted oncogenic functions and promoted NB progression both in vitro and in vivo. Transcriptome analysis revealed that SAPCD2-knockdown led to alterations in genes involved in cell cycle, DNA replication, nuclear division, and spindle formation, which are all key cellular processes modulated by E2F signaling and often deregulated in cancer. In particular, multiple cell cycle-related genes as well as E2F target genes with well-established roles in CIN, including FBXO5, CDC20, and MAD2L1 [23–25], were identified as being regulated by SAPCD2. Our data also indicated that SAPCD2-knockdown led to a decreased E2F activity in NB cells.

Experimental studies show that E2F7 competes with E2F1 in binding to E2F-responsive promoters, and represses the transcriptional activity of target genes [30]. In addition, E2F7 transcription is regulated by E2F1, and E2F1 could be modulated by itself and E2F7. In this way, E2F1 and E2F7 exhibit a dynamic balance [20]. Recent studies reveal that the nuclear import factor KPNA2 and nuclear export factor XPO1 could drive the nucleocytoplasmic shuttling of both E2F1 and E2F7 [20, 31]. Compared to normal tissue controls, however, the relocation of E2F7, but not E2F1, from the nucleus to the cytoplasm is induced by the overexpressed XPO1, and is commonly observed in colorectal cancer, prostate cancer, breast cancer, and HNSCC. The causes of the differences in subcellular distribution of these two XPO1 cargo proteins remain elusive [20]. In our study, the interaction of SAPCD2 and E2F7 was predicted by bioinformatics approach and validated by co-immunoprecipitation assay and immunofluorescence staining, whereas SAPCD2 exhibited no significant interaction with E2F1. An earlier study indicated that SAPCD2 could outcompete PP2A for binding to Axin1, finally blocking Wnt/β-catenin signaling [14]. We supposed that through direct binding to cytoplasmic E2F7, SAPCD2 might inhibit the nuclear import of E2F7 mediated by KPNA2 or other factors, resulting in the imbalance of E2F7 and E2F1 in the nucleus and the derepression of E2F signaling transactivation.

Among the E2F family members, the roles of E2F7 in NB are poorly understood. Recent evidence demonstrated that Selinexor, a clinically available inhibitor of exportin 1 (XPO1), affected the subcellular distribution of E2F7 [20]. Our data suggested that Selinexor could induce nuclear accumulation of E2F7, inhibit E2F signaling and suppress the growth of NB cells. Previous studies revealed that defects in E2F7-dependent transcription might disturb DNA repair and genomic stability, thereby weakening the sensitivity to chemotherapy [32]. In this study, E2F7 mislocalization to the cytoplasm was seen in NB cell line SK-N-BE(2). Interestingly, SK-N-BE(2) have been previously reported to be GSK-J4-resistant NB cell line and not sensitive to low dose (under 1 μM) of GSK-J4 [5]. Our data revealed that Selinexor could sensitize SK-N-BE(2) cells to the low dose of GSK-J4 and enhance NB regression. Our findings are consistent with a very recent study [6] which reported that the E2F pathway could be activated by histone demethylase Jumonji D3 and play important roles in conferring NB cell resistance to GSK-J4, while we provided another potential mechanism regulating E2F signaling in NB cells.

**Fig. 6:**
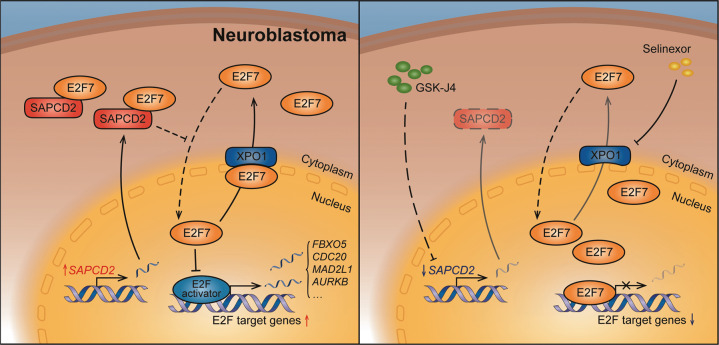
A model depicting the proposed mechanism by which SAPCD2 promotes NB progression. (Left) SAPCD2 binds to E2F7 in the cytoplasm and thereby alters the subcellular distribution of E2F7, derepressing E2F signaling transactivation, and promoting NB progression. XPO1 mediates the nuclear export of E2F7 in NB cells. (Right) GSK-J4 treatment reduces the level of SAPCD2 in NB cells and limits the binding of SAPCD2 to E2F7 in the cytoplasm. Selinexor inhibits the XPO1-dependent nuclear export of E2F7. Nuclear accumulation of E2F7 inhibits the E2F signaling and suppresses the progression of NB cells.

Overall, we demonstrated that SAPCD2 was highly expressed and associated with poor survival of NB. SAPCD2 could bind to E2F7 in the cytoplasm and thereby alter the subcellular distribution of E2F7, derepressing E2F signaling transactivation and promoting NB progression (Fig. 6). Our studies expanded on the previously reported observations on the function of SAPCD2 and added novel mechanistic insights, and might contribute to the development of a therapeutic strategy for clinical NB treatment.

## Materials and methods

### Cell culture

Human NB cell lines (SH-SY5Y, and SK-N-BE(2)) were obtained from the National Collection of Authenticated Cell Cultures (Shanghai, China), and verified by short tandem repeat analysis. Cells were cultured in DMEM/F12 (Thermo Fisher Scientific) supplemented with 10% FBS (Biological Industries, USA) and 1% penicillin-streptomycin (Beyotime, China) at 37 °C with a humidified atmosphere of 5% CO_2_ and examined free of mycoplasma regularly.

### RNA microarray, RNA sequencing, and data analysis

Cells were seeded in six-well plates. Twenty-four hours after seeding, cells were treated with 0.5 μM GSK-J4 (#S7070, Selleck Chemicals) or Selinexor (KTP-330, #S7252, Selleck Chemicals) for 24 h. The medium was removed, and total RNA was extracted with TRIzol (Invitrogen) according to the manufacturer’s protocol. Arraystar Human LncRNA Microarray V4.0 (Arraystar Inc., USA) is designed for the global profiling of human lncRNAs and protein-coding transcripts, which is capable of detecting ~40,173 lncRNAs and 20,730 coding transcripts. RNA isolation, purification, microarray hybridization, array scan, and data analysis were performed by Kangchen Biotech Co., Ltd. (Shanghai, China).

For RNA sequencing, RNA isolation, library preparation, transcriptome sequencing (Illumina NovaSeq 6000), and clean data filtering were carried out by Novogene Bioinformatics Technology Co., Ltd. (Beijing, China). The 150 bp paired-end reads were aligned to the hg38 (Ensembl) genome using HISAT2 software (version 2.2.0). Transcriptome assembly and abundance analysis were performed with StringTie software (version 2.1.2). Differentially expressed genes were identified by R/Bioconductor package DESeq2 [33] with adjusted *P* value <0.05 and absolute log_2_(fold change) >1. Gene Ontology (GO) enrichment analysis was performed using Metascape (http://metascape.org/gp/). Gene set enrichment analysis (GSEA) was conducted using R/Bioconductor package clusterProfiler [34] with Hallmarks gene sets (version 7.1) from the Molecular Signatures Database (MSigDB). Transcription factor (TF) binding motifs enrichment was performed using R/Bioconductor package RcisTarget [35]. TF annotation collection (hg38_refseq-r80_10kb_up_and_down_tss.mc9nr) was obtained from cisTarget database (https://resources.aertslab.org/cistarget/).

### Data mining of public datasets

Gene expression and copy number alteration (CNA) data were obtained from The Cancer Genome Atlas (TCGA) database (https://portal.gdc.cancer.gov/, with project TARGET-NBL, and TCGA sub-projects containing both normal and tumor samples) and the Therapeutically Applicable Research to Generate Effective Treatments (TARGET) database (https://target-data.nci.nih.gov/Public/NBL/WXS/). Transcripts were quantified with StringTie software (version 2.1.2) in TPM (transcripts per million mapped transcripts). Chromosome instability index (CIN) scores were devised in a previous study [18] to measure the degree of CNAs across the entire genome of a tumor, taking into account both the total regions of the chromosome that are altered in a tumor as well as the amplitude of these alterations. The correlations between transcript levels and CIN scores were calculated and visualized using R/Bioconductor packages ggpubr and ggplot2.

### Luciferase reporter assay

Dual-luciferase reporter vector psiCHECK-2 (Promega) carrying either the wild-type (WT) E2F motifs or a mutant version (Mut), and the vector pLVX-EF1α-IRES-puro (Clontech) carrying FLAG-tagged SAPCD2 were constructed by General Biosystems (Anhui, China). pLVX-EF1α-IRES-puro carrying HA-tagged E2F7 was constructed by Tsingke Biological Technology (Beijing, China). Recombinant constructs were verified by Sanger sequencing. Cells were transfected using Lipofectamine 3000 (Thermo Fisher Scientific). After 48 h of transfection, the luciferase activity was measured by the Dual-Luciferase Reporter Assay System (Promega).

### Statistical analysis

Statistical analysis was performed using GraphPad Prism version 8.0.2 (GraphPad Software, Inc., USA). Differences were considered statistically significant when *p* < 0.05 (*p < 0.05, **p < 0.01).

## Supplementary information

Supplementary materials and methods

Supplementary figure and table legends

Supplementary Fig. S1

Supplementary Fig. S2

Supplementary Fig. S3

Supplementary Fig. S4

Supplementary Fig. S5

Supplementary Table S1

Supplementary Table S2

Original Western Blots

Author Contribution Statement

AJ-checklist

## Data Availability

RNA microarray and RNA-seq data supporting the results of the current study have been deposited in the GEO database (https://www.ncbi.nlm.nih.gov/geo, with accession code GSE180601 and GSE186245). Public datasets are available from the GEO database (https://www.ncbi.nlm.nih.gov/geo) with accession codes GSE16476, GSE62564, and GSE147635. The copy number and genetic variation data are available from the Oncogenomics database (https://pob.abcc.ncifcrf.gov/cgibin/JK) and cBioPortal for Cancer Genomics (http://cbioportal.org) with search term “SAPCD2”. The datasets used and/or analyzed during the current study are available from the corresponding author on reasonable request. Original western blots are attached as Supplemental materials.
