# Supplementary material for: SAPCD2 promotes neuroblastoma progression by altering the subcellular distribution of E2F7
Source: Cell Death Dis. 2022 Feb 23;13(2):174. doi: 10.1038/s41419-022-04624-z (PMC8866461; doi:10.1038/s41419-022-04624-z)
Supplement: Supplementary file 1 — Supplementary materials and methods [file 41419_2022_4624_MOESM1_ESM.docx]

**Supplementary materials and methods**

**Western blot analysis**

Protein lysates of cells were isolated by RIPA buffer containing 1 mM PMSF and 1% phosphatase inhibitor cocktail. Equal amounts of protein (20 μg) were separated on SDS PAGE and transferred onto PVDF membranes for western blot analysis using antibodies specific for GAPDH (MAB374, Millipore), SAPCD2 (HPA044154, Atlas Antibodies), PARP (#9542, Cell Signaling Technology), Caspase-3 (#9662, Cell Signaling Technology), Cleaved caspase-3 (#9664, Cell Signaling Technology), E2F1 (66515-1-Ig, Proteintech), E2F4 (10923-1-AP, Proteintech) and E2F7 (24489-1-AP, Proteintech). Chemiluminescent HRP substrate (Millipore, USA) was used to detect the antibody-antigen complexes.

**Immunohistochemistry and analysis**

The NB tissue microarray (NB642a, Biomax, USA) was stained with antibodies specific for SAPCD2 (HPA044154, Atlas). UltraSensitive SP (Mouse/Rabbit) IHC Kit and DAB Plus Kit (MXB Biotechnologies, China) were used following the manufacturer's protocol. The staining results of each tissue section were observed under the Olympus BX41 imaging system, and scored by multiplying the percentage of positive cells (P) by the intensity (I).

**Knockdown or overexpression of genes**

The sequences of shRNA were synthesized by IGE Biotechnology, Ltd. (Guangzhou, China) and listed below. Lentiviral packaging was performed as previously described^1^. Lentiviral medium, if necessary, was concentrated by PEG-8000 (Beyotime, China) precipitation. Cells were infected with lentiviral particles in the presence of polybrene (Sigma-Aldrich). After screening with puromycin (Sigma-Aldrich), stable knockdown or overexpression cells were established.

| **Gene name** | **Sequence** |
| --- | --- |
| sh-SAPCD2 #1 | CCGGACCTCTGGATTCCACCTTCATCTCGAGATGAAGGTGGAATCCAGAGGTTTTTTGAATT |
| sh-SAPCD2 #2 | CCGGGCTGCAGGGTTTGGAGATGATCTCGAGATCATCTCCAAACCCTGCAGCTTTTTGAATT |

**Cell viability assay**

Cells (2×10^3^ per well) were seeded in 96-well plates, and cell viability was detected by cell counting kit-8 (CCK8) assay (Dojindo Molecular Technologies, Japan) as manufacturer’s instructions.

**Colony formation**

Cells were seeded in 6-well plates at a density of 2×10^3^ per well and incubated for 21 days. Cells were washed with PBS, fixed in ice-cold methanol and stained with 0.1% crystal violet. Colonies were imaged and counted.

**Transwell migration assay**

Transwell plates (24-well, pore size 8 μm, Corning) were used for the transwell assay. 1×10⁵ NB cells were harvested in 100 μl of serum-free culture medium and added into the upper chamber.600 μl of 15% fetal bovine serum medium was placed into the bottom compartment of the chamber as a source of chemo-attractant. After 48 h culturing, the cells that crossed the inserts were fixed with 4% formaldehyde and stained with Wright’s-Giemsa. Attached cells were photographed and counted under the microscope.

**Cell apoptosis and cell cycle analysis**

For cell apoptosis analysis, cells were harvested by trypsin solution without EDTA, washed, and stained using Annexin V-FITC kit (Miltenyi Biotec). For cell cycle analysis, cells were harvested, washed, and fixed in ice-cold 70% ethanol overnight at 4℃. The next day, cells were incubated with propidium iodide/RNase A staining solution (Sigma-Aldrich) at room temperature for 20 minutes. Samples were analyzed by flow cytometry (Beckman Gallios, Germany), and cell cycle distribution was analyzed using FlowJo v10 software (Tree Star, USA).

***In Vivo* Xenografts**

Four-week-old female BALB/c nude mice (n=6 per group) received the subcutaneous injection at the dorsal flanks with NB cells (2×10^6^ cells for each mouse) suspended in 100 μl Matrixgel Matrix (Corning). For the study of SAPCD2 knockdown, NB cells were stably transduced with scramble shRNA or shRNA targeting SAPCD2. The alteration in volume of tumors was recorded. Mice were sacrificed when the largest tumors were ∼15 mm in diameter. Tumors were dissected, weighted, and analyzed. The Animal Care and Use Committee of Soochow University approved all animal studies, which were performed in accordance with the guidelines for animal care.

**RNA isolation and qRT-PCR**

Total RNA was prepared by the RNeasy Mini Kit (Qiagen). Reverse transcription was carried out using Reverse Transcription System (Promega). For PCR, LightCycler 480 SYBR Green I Master mix (Roche) was applied on a Light cycler 480 Real-Time System (Roche). Expression was determined by 2^-△△Ct^ method and GAPDH expression as an internal reference. The qPCR primers used were synthesized by Tsingke Biological Technology (Beijing, China) and listed below.

| **Gene name** |  | **Sequence** |
| --- | --- | --- |
| SAPCD2 | F | GCAGACCATCCTCATGCTGAA |
|  | R | CGTGATGCGCTCACTCTTCT |
| E2F1 | F | ACGTGACGTGTCAGGACCT |
|  | R | GATCGGGCCTTGTTTGCTCTT |
| E2F2 | F | CGTCCCTGAGTTCCCAACC |
|  | R | GCGAAGTGTCATACCGAGTCTT |
| E2F3 | F | CGGCAGCCTCCTCTACAC |
|  | R | AGCTCCAGCCTTCGCTTT |
| AURKB | F | CTCCTACCCCTGGCCCTAC |
|  | R | AGGCTCTTTCCGGAGGACT |
| PLK1 | F | CACAGTGTCAATGCCTCCA |
|  | R | TTGCTGACCCAGAAGATGG |
| BIRC5 | F | GCCCAGTGTTTCTTCTGCTT |
|  | R | CCGGACGAATGCTTTTTATG |
| CDC20 | F | CTGTCTGAGTGCCGTGGAT |
|  | R | TCCTTGTAATGGGGAGACCA |
| KIF2C | F | AGGAGCATCTGGTTAACTCTGC |
|  | R | TCTGCCCAGAGGTTCTGC |
| MAD2L1 | F | CGCGTGCTTTTGTTTGTGT |
|  | R | GCTGTTGATGCCGAATGAG |
| SMC4 | F | GAGGAACACACTAGAAAAATTTACTGC |
|  | R | GGACATTCAAATATTCTTCTTGCTCTA |
| FBXO5 | F | CTACACCCCTGCATATTTGGAAG |
|  | R | TGACCCAATACATGACAGCCTT |
| CDK1 | F | AAACTACAGGTCAAGTGGTAGCC |
|  | R | TCCTGCATAAGCACATCCTGA |
| CDK2 | F | CCAGGAGTTACTTCTATGCCTGA |
|  | R | TTCATCCAGGGGAGGTACAAC |
| CCNB1 | F | TTGGGGACATTGGTAACAAAGTC |
|  | R | ATAGGCTCAGGCGAAAGTTTTT |

**Co-immunoprecipitation**

Co-immunoprecipitation was performed as previously described^1^, using 10 µg of antibodies for SAPCD2 (HPA044154, Atlas) or E2F7 (24489-1-AP, Proteintech). After eluting from bead-bound immunocomplexes, protein levels were determined by western blotting.

**Immunofluorescence**

Cells grown on confocal dish were fixed with 1% paraformaldehyde, incubated with 0.5% Triton X-100 in PBS, blocked with 5% BSA and treated with antibodies specific for SAPCD2 (HPA044154, Atlas), FLAG (F1804, Sigma-Aldrich) or E2F7 (24489-1-AP, Proteintech) at 4℃ overnight. The next day, cells were treated by Alexa Fluor 488 goat anti-mouse IgG or Alexa Fluor 594 goat anti-rabbit IgG (Jackson ImmunoResearch), and stained by DAPI Fluoromount-G (#0100-20, SouthernBiotech). The images were photographed under confocal microscopy.

**Reference**

1. Monteverde T, Sahoo S, La Montagna M, Magee P, Shi L, Lee D, et al. CKAP2L Promotes Non-Small Cell Lung Cancer Progression through Regulation of Transcription Elongation. Cancer Res 2021, 81(7): 1719-1731.
