# Supplementary material for: SAPCD2 promotes neuroblastoma progression by altering the subcellular distribution of E2F7
Source: Cell Death Dis. 2022 Feb 23;13(2):174. doi: 10.1038/s41419-022-04624-z (PMC8866461; doi:10.1038/s41419-022-04624-z)
Supplement: Supplementary file 2 — Supplementary figure and table legends [file 41419_2022_4624_MOESM2_ESM.docx]

**Supplementary figure and table legends**

**Supplementary Fig. S1.** The expression of SAPCD2 in ganglioneuroma (GN) tissue, neuroblastoma (NB) cell lines and NB tissue. RNA-Seq data are derived from GSE14340. RPKM, Reads Per Kilobase per Million mapped reads. Unpaired two-sided *t* test for analysis, data are shown as mean ± SD (error bars).

**Supplementary Fig. S2.** The analysis of diverse datasets from The Cancer Genome Atlas (TCGA) indicated a positive correlation between SAPCD2 transcript levels and CIN score in multiple tumors. Pearson’s correlation coefficient for analysis in this figure.

**Supplementary Fig. S3.** GSEA analysis plot of deregulated genes in SAPCD2-knockdown cells with SAPCD2-correlated gene sets derived from 88 (GSE16476) and 498 specimens (GSE62564). NES, Normalized Enrichment Score. NOM *p* value, adjusted *p* value.

**Supplementary Fig. S4.** Immunofluorescence confocal images showing the subcellular distribution of E2F7 in SK-N-BE(2) cells exposed to the different reagents as indicated. Scale bars, 10μm.

**Supplementary Fig. S5.** A-B. Several E2F-targeted genes associated with CIN were downregulated in SAPCD2-knockdown SK-N-BE(2) cells and predicted poor prognosis in NB patients. C. The analysis of diverse public datasets indicated a significant positive correlation between SAPCD2 transcript levels and these E2F-targeted genes across multiple tumors. Log-rank test for analysis in B. Pearson’s correlation coefficient in C.

**Supplementary Table. S1.** Gene Set Enrichment Analysis (GSEA) with Hallmark signatures.

**Supplementary Table. S2.** motif enrichment analysis by cisTarget to identify upstream transcription factors that might target the DEGs or the genes enriched in GSEA set HALLMARK_E2F_TARGETS in SAPCD2-knockdown SK-N-BE(2) cell.

**Supplementary File.** Original Western Blots.
