# Supplementary figures and images for: SAPCD2 promotes neuroblastoma progression by altering the subcellular distribution of E2F7
Source: Cell Death Dis. 2022 Feb 23;13(2):174. doi: 10.1038/s41419-022-04624-z (PMC8866461; doi:10.1038/s41419-022-04624-z)

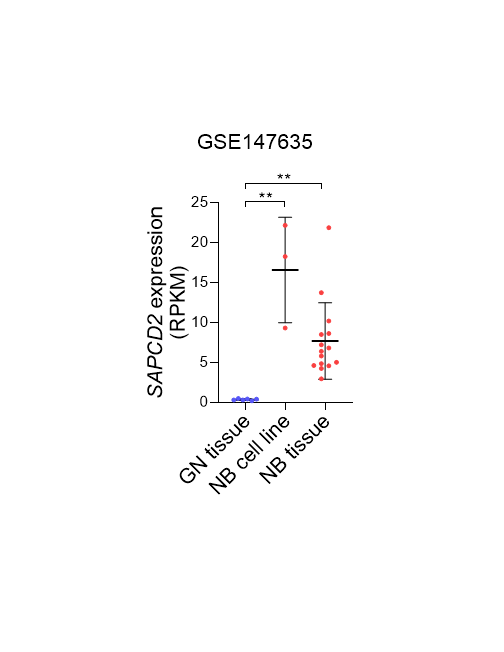

Supplement: Supplementary file 3 — Supplementary Fig. S1 [file 41419_2022_4624_MOESM3_ESM.tif]

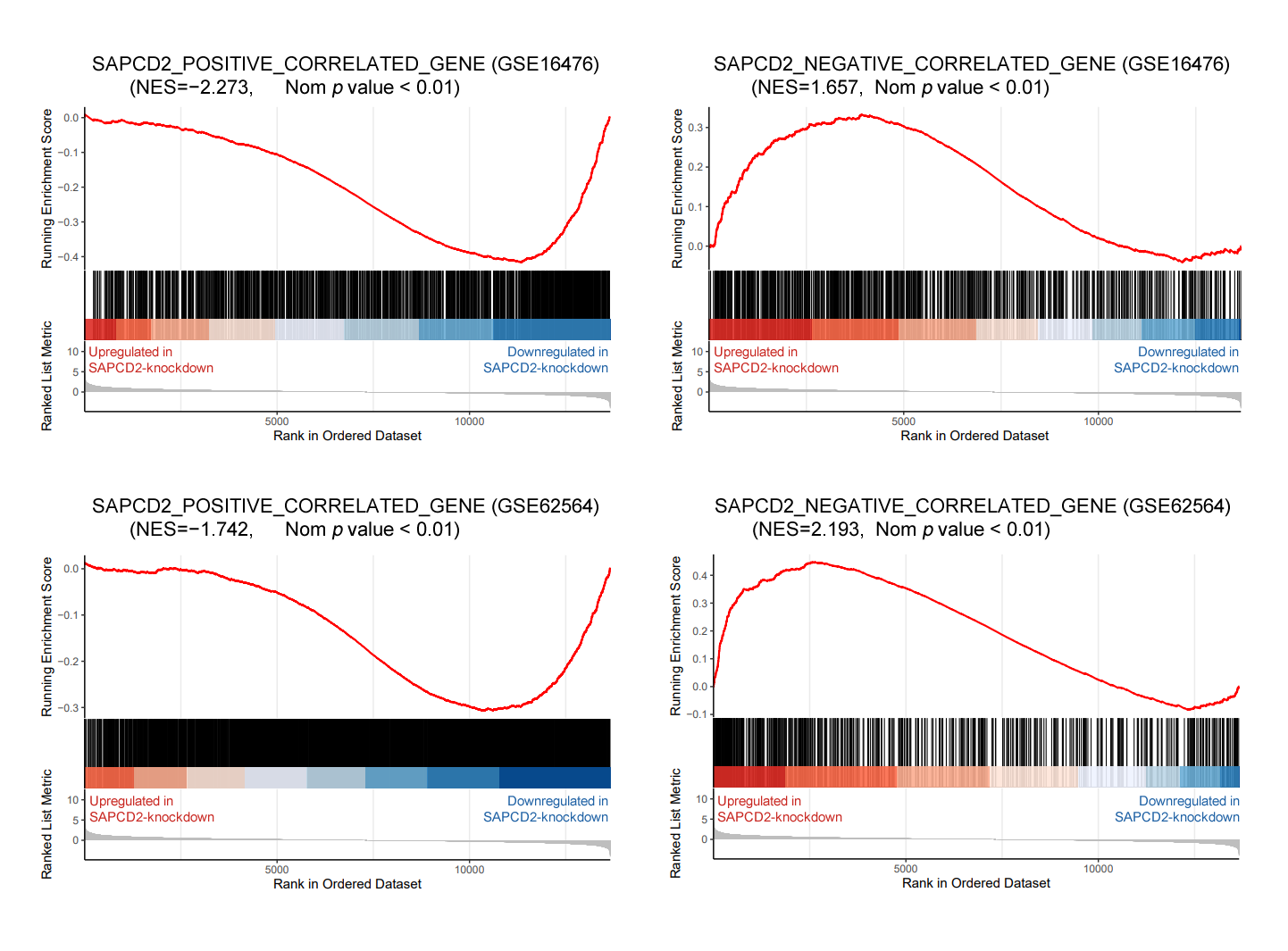

Supplement: Supplementary file 5 — Supplementary Fig. S3 [file 41419_2022_4624_MOESM5_ESM.tif]

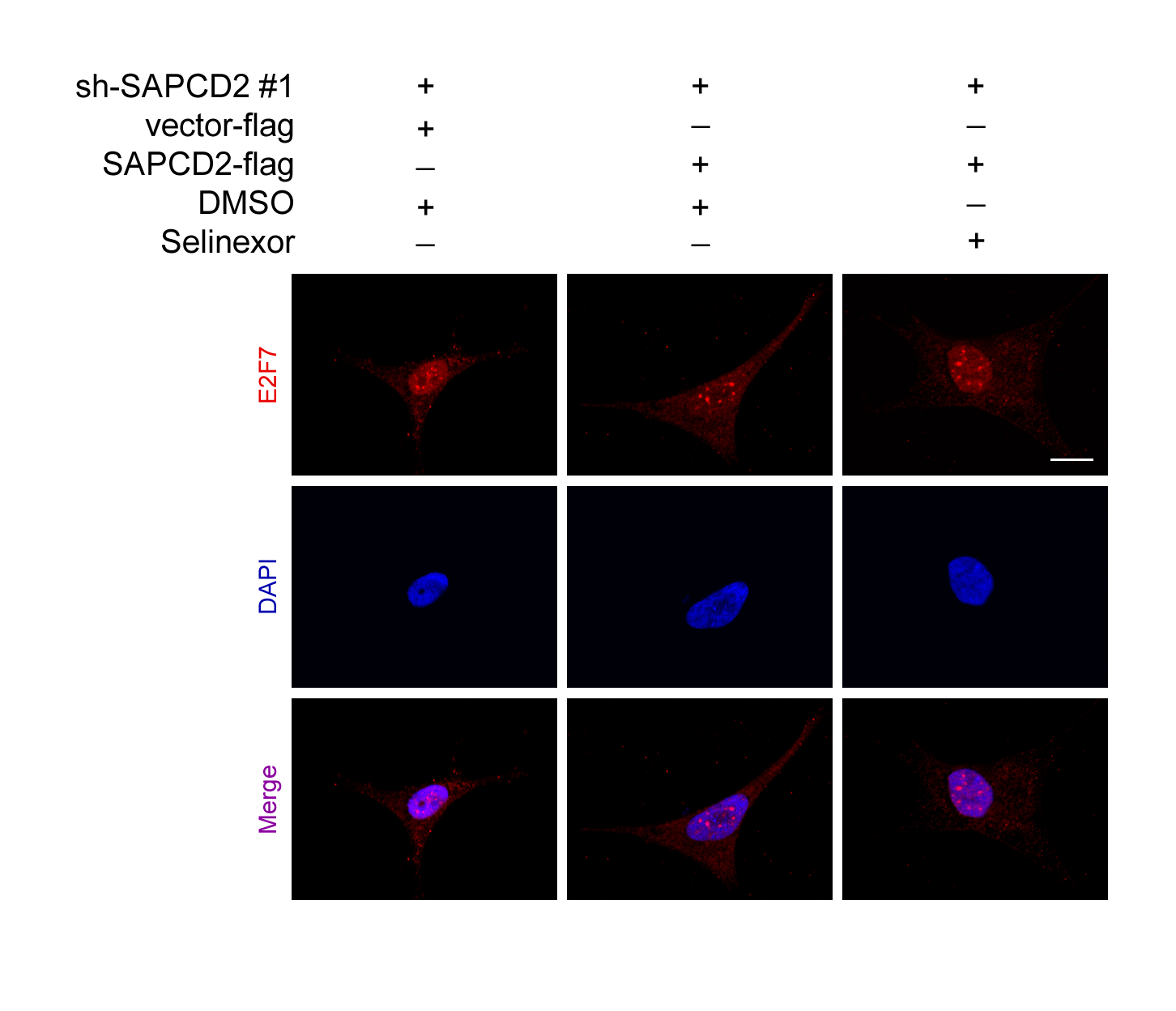

Supplement: Supplementary file 6 — Supplementary Fig. S4 [file 41419_2022_4624_MOESM6_ESM.tif]

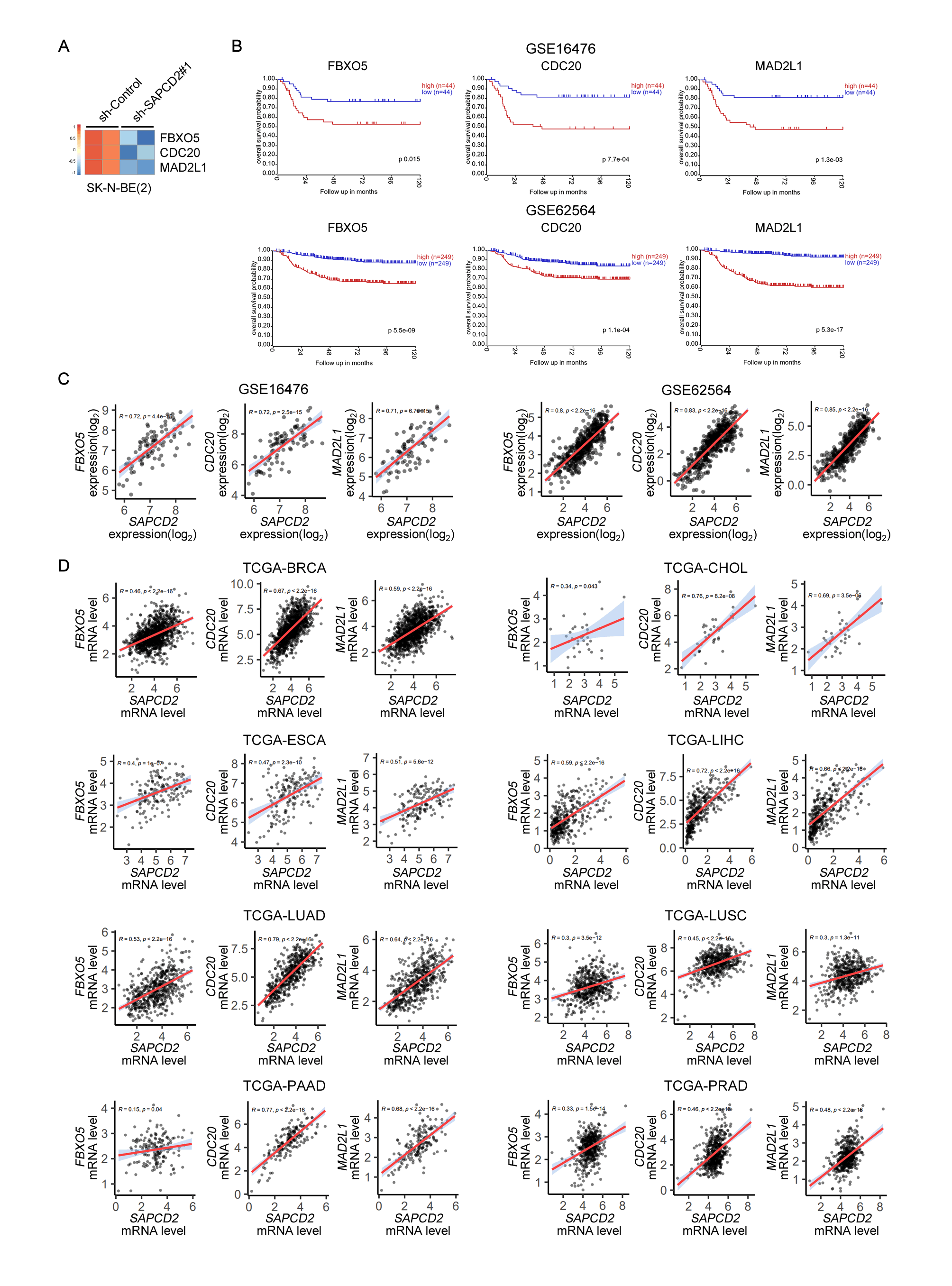

Supplement: Supplementary file 7 — Supplementary Fig. S5 [file 41419_2022_4624_MOESM7_ESM.tif]
