# Supplementary material for: SAPCD2 promotes neuroblastoma progression by altering the subcellular distribution of E2F7
Source: Cell Death Dis. 2022 Feb 23;13(2):174. doi: 10.1038/s41419-022-04624-z (PMC8866461; doi:10.1038/s41419-022-04624-z)
Supplement: Supplementary file 8 — Supplementary Table S1 [file 41419_2022_4624_MOESM8_ESM.pdf]

Supplementary Table 2 - Gene Set Enrichment Analysis (GSEA) with Hallmark signatures showed significant enrichment

ID	setSize	enrichmer	NES	pvalue	p.adjust	qvalues	rank	leading_edge	core_enrichment
HALLMARK_E2F_TARGETS	199	-0.6911	-3.2224	0.002915	0.008098	0.003069	2597	tags=67%, list=19%, signal=55%	TIPIN/CTCF/CDKN3/ILF3/H2AZ1/SYNCRIP/DONSON/EZH2/CDC25A/ANP32E/MLH1/TACC3/DNMT1/RAD51C/RAN/RAD21/SPAG5/PRKDC/NUP153/RPA1/BRMS1L/WDR90/NCAPD2/CENPM/SRSF2/MSH2/NUP107/CTPS1/LMNB1/XPO1/ZW10/CNOT9/DEPDC1/UBE2T/SSRP1/PLK1/MCM2/NUP205/GINS3/CBX5/TUBB/SMC6/RFC1/NUDT21/STAG1/CDCA3/MRE11/DCLRE1B/CSE1L/TMPO/CDC20/POLE/PSIP1/MELK/CCNB2/DIAPH3/TP53/POLD1/CKS1B/BIRC5/EXOSC8/USP1/KIF2C/RACGAP1/CHEK1/BUB1B/ESPL1/ING3/RPA3/HMGB3/NBN/BARD1/TRIP13/CDCA8/KIF22/PRIM2/NASP/HELLS/HMGB2/CHEK2/SPC24/PAN2/H2AX/UBR7/RAD51AP1/DEK/KIF18B/MCM6/MCM3/CDK1/DSCC1/RRM2/MYBL2/PTTG1/SMC4/MCM5/TIMELESS/MCM4/PCNA/AURKB/MMS22L/HMGA1/KIF4A/POLD3/PSMC3IP/TK1/SUV39H1/SPC25/RFC3/PLK4/DLGAP5/RFC2/ORC6/SMC1A/CIT/MKI67/POLA2/GINS1/TOP2A/SHMT1/CENPE/CDKN1A/MAD2L1/ASF1B/LIG1/TCF19/HMMR/DUT/ATAD2/BRCA1/GINS4/BRCA2/E2F8
HALLMARK_G2M_CHECKPOINT	196	-0.58345	-2.71589	0.002865	0.008098	0.003069	3538	tags=62%, list=26%, signal=47%	NUMA1/HNRNPU/CENPA/UBE2S/DBF4/ABL1/PAFAH1B1/KATNA1/HNRNPD/DKC1/E2F3/KIF23/CDC27/MEIS1/PRMT5/NEK2/SFPQ/SRSF1/PDS5B/STMN1/SRSF10/RPA2/TRA2B/INCENP/CTCF/CDKN3/ILF3/ATF5/H2AZ1/ORC5/SYNCRIP/EZH2/CDC25A/TACC3/SS18/MNAT1/RAD21/SMARCC1/CHAF1A/PBK/SRSF2/E2F1/NSD2/LMNB1/XPO1/UBE2C/PLK1/MCM2/CCNA2/BUB1/RAD54L/STAG1/TPX2/TMPO/MAP3K20/H2AZ2/CDC20/EFNA5/POLE/SNRPD1/CCNB2/CKS1B/PRC1/BIRC5/KIF2C/RACGAP1/CHEK1/TROAP/ESPL1/POLQ/HMGB3/BARD1/KIF22/PRIM2/CCNF/FBXO5/MT2A/BUB3/NASP/EXO1/E2F2/CASP8AP2/H2AX/RBL1/MCM6/CENPF/MCM3/CDC7/CDK1/MYBL2/SMC2/NDC80/PTTG1/SMC4/MCM5/AURKB/HMGA1/KIF4A/KIF11/TRAIP/NUSAP1/SUV39H1/CDC45/PLK4/ORC6/NOTCH2/SMC1A/TTK/MKI67/GINS2/STIL/KNL1/POLA2/KIF20B/TOP2A/KIF15/CDC6/CENPE/HMGN2/MAD2L1/HMMR/BRCA2
HALLMARK_MITOTIC_SPINDLE	196	-0.39741	-1.8499	0.002865	0.008098	0.003069	1866	tags=28%, list=14%, signal=24%	ALMS1/LMNB1/SYNPO/HOOK3/PLK1/PCM1/ECT2/BUB1/RFC1/EPB41L2/TPX2/CENPJ/TUBGCP5/PLEKHG2/CEP192/CCNB2/ARHGAP29/PRC1/BIRC5/ANLN/KIF2C/RACGAP1/ESPL1/ARHGAP10/KIF22/FBXO5/SASS6/LATS1/ITSN1/CENPF/CDK5RAP2/CDK1/MYO1E/MID1/NDC80/PDLIM5/SMC4/SORBS2/KIF4A/KIF11/NUSAP1/DLGAP5/NOTCH2/SMC1A/TTK/KNTC1/KIF20B/TOP2A/KIF15/CENPE/WASF1/WASF2/BRCA2/CDC42EP1
HALLMARK_SPERMATOGENESIS	83	-0.36539	-1.49297	0.01519	0.027614	0.010464	3660	tags=52%, list=27%, signal=38%	SNAP91/CCT6B/PHF7/SIRT1/SPATA6/MLLT10/CAMK4/DBF4/MLF1/CSNK2A2/VDAC3/NEK2/COIL/MTOR/TSN/ZC3H14/IFT88/HSPA2/CDKN3/RPL39L/ARL4A/SCG3/ZC2HC1C/EZH2/PSMG1/AGFG1/IDE/BUB1/TOPBP1/CCNB2/KIF2C/RFC4/NPHP1/CHRM4/CDK1/GRM8/TTK/DMC1/CCNA1/ACRBP/NCAPH/ACRV1/CNIH2
HALLMARK_DNA_REPAIR	144	-0.31401	-1.40799	0.007937	0.016534	0.006266	2106	tags=30%, list=15%, signal=26%	RPA2/SUPT4H1/SNAPC5/ELOA/ERCC1/POLR2G/GTF2H3/FEN1/UMPS/ERCC8/RAD52/NT5C3A/ERCC5/AK3/SRSF6/SSRP1/POLR3C/SF3A3/TK2/DDB2/POM121/NUDT21/AAAS/NELFB/TP53/POLD1/ADA/RPA3/TAF6/POLA1/RFC4/RFC5/ZWINT/RAD51/PCNA/POLD3/TYMS/RFC3/RFC2/ZNRD1/POLA2/LIG1/DUT/PRIM1
HALLMARK_MYC_TARGETS_V1	199	-0.29997	-1.39865	0.014577	0.027614	0.010464	3011	tags=25%, list=22%, signal=20%	NPM1/XRCC6/HNRNPA2B1/SRSF3/SRSF1/HPRT1/MCM7/RNPS1/TRA2B/ILF2/IARS1/H2AZ1/SYNCRIP/PRPS2/YWHAE/RAN/SMARCC1/SRSF2/CTPS1/NCBP1/XPO1/SET/MCM2/CCNA2/PRPF31/CDC20/SNRPD1/UBE2E1/UBA2/RRM1/USP1/HNRNPA3/GLO1/BUB3/RFC4/DEK/SSB/MCM6/LSM2/SF3A1/MCM5/MCM4/LDHA/PCNA/TYMS/CDC45/SRM/CDK2/MAD2L1/PRPF31/DUT/PRPF31
HALLMARK_APICAL_JUNCTION	147	0.325837	1.38383	0.014263	0.027614	0.010464	4398	tags=43%, list=32%, signal=29%	ICAM5/CDH15/MMP9/ITGA3/KCNH2/CNN2/CD276/ICAM2/VASP/PIK3CB/SIRPA/ATP1A3/NECTIN2/CDH4/STX4/RAC2/CLDN4/TRO/PIK3R3/NRXN2/ADAM15/COL16A1/HRAS/PBX2/CADM2/VAV2/MMP2/MDK/SDC3/ARHGEF6/CD99/ACTN2/SLC30A3/ADAM9/PARD6G/PCDH1/NLGN2/TAOK2/SORBS3/CLDN18/PLCG1/CDH3/YWHAH/TSPAN4/SPEG/BMP1/PKD1/PFN1/THY1/THBS3/GNAI1/ITGA2/SRC/RRAS/INSIG1/LAYN/BAIAP2/MAP4K2/SYMPK/PTPRC/SHC1/ITGB1/MADCAM1
HALLMARK_ALLOGRAFT_REJECTION	94	0.362396	1.435062	0.015464	0.027614	0.010464	2800	tags=29%, list=21%, signal=23%	RPL3L/ZAP70/MMP9/CD4/TAP2/CD40/CTSS/CFP/RPS9/HLA-A/PSMB10/IL12A/ST8SIA4/CSK/GALNT1/BCL3/IL2RB/CCND3/FAS/INHBA/B2M/F2R/TAPBP/EIF4G3/CDKN2A/RPS19/AKT1
HALLMARK_MTORC1_SIGNALING	192	0.325262	1.438292	0.006154	0.013891	0.005264	2245	tags=24%, list=16%, signal=20%	STC1/SLC7A11/POLR3G/BTG2/TUBA4A/CTH/EGLN3/SQSTM1/CFP/HK2/EDEM1/TRIB3/G6PD/GLRX/TES/XBP1/DDIT3/MAP2K3/IFI30/SLC9A3R1/SLC6A6/SLC7A5/MLLT11/NFKBIB/SLC1A5/FKBP2/RIT1/SDF2L1/SLC2A3/PIK3R3/CXCR4/TFRC/PPP1R15A/CCNG1/BHLHE40/UFM1/SERP1/SERPINH1/GCLC/HSPA5/M6PR/ATP2A2/RAB1A/USO1/DDIT4/SSR1

HALLMARK_COMPLEMENT	126	0.353146	1.465091	0.00639	0.013891	0.005264	2746	tags=33%, list=20%, signal=27%	MAFF/C1S/MMP14/CTSS/MMP15/CASP4/CEBPB/CASP9/HPCAL4/EHD1/CLU/CTSD/LIPA/PRKCD/PRSS3/LRP1/DGKG/DOCK4/CDK5R1/RCE1/STX4/COL4A2/ANG/CTSL/CTSB/CALM3/CASP7/LAP3/HSPA1A/PPP4C/HSPA5/APOC1/PRCP/SPOCK2/CASP3/DYRK2/ACTN2/ADAM9/PDP1/IRF2/PLAT/CD46
HALLMARK_XENOBIOTIC_METABOLISM	141	0.349921	1.466684	0.004934	0.011748	0.004452	3555	tags=38%, list=26%, signal=28%	AKR1C3/CYP1A1/APOE/ABCC3/GSTA3/UPP1/ABCC2/GABARAPL1/ASL/EPHA2/DDAH2/SPINT2/PSMB10/FAH/BLVRB/HES6/SLC6A6/FBP1/SLC1A5/CBR1/PAPSS2/CROT/COMT/DCXR/SLC12A4/AKR1C2/NINJ1/SMOX/ACO2/ALDH2/TMEM176B/ACOX3/NQO1/ARG2/GCLC/ATP2A2/DHRS7/FAS/CYP2J2/MAN1A1/IDH1/GCNT2/PTGES/POR/CYP2E1/CYP27A1/PEMT/TMBIM6/DDT/IL1R1/CNDP2/GSR/TPST1
HALLMARK_HEME_METABOLISM	161	0.34668	1.493042	0.004769	0.011748	0.004452	2669	tags=27%, list=20%, signal=22%	ACP5/P4HA2/SLC7A11/FTCD/BTG2/SLC6A8/PPP2R5B/SLC30A1/ANK1/ALDH1L1/MAP2K3/TMEM9B/BLVRB/CLCN3/BMP2K/GLRX5/BACH1/DAAM1/SLC11A2/ABCB6/KLF3/CTSB/SMOX/CDR2/ACSL6/TFRC/HTATIP2/RCL1/SLC25A38/CPOX/ADIPOR1/CCND3/RNF19A/GCLC/FN3K/MARCHF2/ENDOD1/MINPP1/TNRC6B/HTRA2/SNCA/AGPAT4/HAGH/ATP6V0A1
HALLMARK_OXIDATIVE_PHOSPHORYLATION	198	0.337291	1.500489	0.001522	0.005659	0.002145	5116	tags=47%, list=37%, signal=30%	NDUFA3/ATP6V0E1/ATP6V0B/SLC25A20/ATP6AP1/COX10/MRPL34/TIMM17A/VDAC1/ATP6V1F/ATP6V0C/PDHX/GPX4/POLR2F/SLC25A6/HCCS/ACO2/TIMM13/CASP7/NDUFA1/GRPEL1/MRPS12/ATP6V1G1/BAX/UQCRFS1/ECHS1/OAT/PMPCA/NDUFB2/ACADVL/HTRA2/NQO2/TIMM8B/HSD17B10/ACAA1/ATP1B1/SLC25A4/CPT1A/PDP1/NDUFB8/ATP6V1H/IDH1/UQCR11/OXA1L/POR/NDUFA2/ISCU/ATP5ME/HSPA9/TIMM10/ETFB/RHOT2/RHOT1/ATP5MC3/SLC25A3/CYC1/IDH3A/TCIRG1/AFG3L2/ATP6V1C1/ALDH6A1/ATP6V1E1/COX5A/ATP5F1D/ETFA/SLC25A12/NDUFS6/TIMM50/CYCS/MDH2/ACAT1/COX5B/MPC1/SUCLA2/ATP5MF/COX7A2/MRPL15/LDHB/NDUFAB1/COX17/NDUFB7/NDUFC2/NDUFA7/NDUFB1/AIFM1/DLD/COX6A1/NDUFV1/NDUFA8/COX6B1/ACAA2/MFN2/ATP5F1A
HALLMARK_UNFOLDED_PROTEIN_RESPONSE	112	0.376345	1.535984	0.003333	0.008772	0.003324	3875	tags=40%, list=28%, signal=29%	WIPI1/DNAJB9/KDELR3/HERPUD1/PDIA5/EDEM1/SRPRA/CEBPB/XBP1/BAG3/TSPYL2/ATF3/ARFGAP1/SLC7A5/EXOSC4/SLC30A5/CHAC1/VEGFA/ERN1/EXOSC5/SERP1/YIF1A/HSPA5/ALDH18A1/NABP1/DDIT4/SSR1/TUBB2A/EEF2/PREB/EIF4G1/WFS1/ATP6V0D1/NHP2/HSPA9/DNAJC3/DNAJA4/RRP9/IMP3/EXOC2/EIF4A2/PSAT1/SEC31A/GOSR2/XPOT
HALLMARK_ESTROGEN_RESPONSE_EARLY	148	0.360847	1.536196	0.001582	0.005659	0.002145	2495	tags=31%, list=18%, signal=26%	HSPB8/DHRS2/DEPTOR/CISH/FOS/IGF1R/KRT19/RET/FAM102A/XBP1/BLVRB/PLAAT3/SLC9A3R1/MREG/SLC7A5/NBL1/HES1/PAPSS2/MLPH/TTC39A/ALDH3B1/TPBG/TIAM1/RAB31/CCND1/KAZN/OLFML3/RRP12/MYBBP1A/MAPT/TOB1/CA12/RPS6KA2/BHLHE40/UNC119/SLC19A2/RARA/SCARB1/ENDOD1/TSKU/LRIG1/PPIF/UGCG/SLC22A5/SLC39A6/ABHD2
HALLMARK_GLYCOLYSIS	175	0.360617	1.578898	0.001572	0.005659	0.002145	2438	tags=29%, list=18%, signal=24%	IER3/STC1/P4HA2/KDELR3/CHPF2/CTH/COL5A1/ISG20/EGLN3/HK2/PGAM2/IRS2/CHST1/G6PD/GLRX/GFPT1/QSOX1/TSTA3/NOL3/CACNA1H/PMM2/EFNA3/RRAGD/SLC16A3/TPBG/PFKP/ANG/PYGL/NT5E/CHPF/IDUA/ABCB6/VEGFA/DPYSL4/HS6ST2/COPB2/CXCR4/MERTK/IL13RA1/PLOD1/ADORA2B/GCLC/GNE/HSPA5/DDIT4/SDC3/ALG1/GMPPA/B3GAT3/B4GALT4
HALLMARK_UV_RESPONSE_UP	130	0.39778	1.647139	0.001631	0.005659	0.002145	2655	tags=36%, list=19%, signal=29%	CYP1A1/MMP14/BTG2/TUBA4A/KCNH2/SLC6A8/FOS/SQSTM1/RET/CLCN2/TYRO3/NAT1/TST/CDKN1C/RHOB/ATF3/IGFBP2/GRINA/PRKCD/MSX1/JUNB/ABCB1/SELENOW/FURIN/ATP6V1F/CDC34/NR4A1/NPTX2/SPR/E2F5/GLS/TFRC/CHKA/GRPEL1/CLTB/MGAT1/CCND3/PPP1R2/DLG4/BCL2L11/GPX3/CYB5R1/PPIF/CASP3/BAK1/ACAA1/SLC25A4
HALLMARK_IL2_STAT5_SIGNALING	143	0.391695	1.655607	0.001605	0.005659	0.002145	2434	tags=32%, list=18%, signal=27%	MAFF/IFITM3/RGS16/SYNGR2/CISH/GADD45B/COL6A1/GABARAPL1/IGF1R/SPRY4/HK2/TNFRSF1B/XBP1/ST3GAL4/RNH1/FAH/PTGER2/CDKN1C/RHOB/NRP1/KLF6/SYT11/ETV4/SLC1A5/TTC39B/RRAGD/ABCB1/FURIN/TIAM1/NT5E/GPX4/SLC2A3/ALCAM/IGF2R/TNFRSF21/BHLHE40/IL2RB/CCND3/ODC1/PLEC/CKAP4/PUS1/LRIG1/ITGA6/APLP1/CASP3
HALLMARK_APOPTOSIS	131	0.407468	1.690342	0.001623	0.005659	0.002145	3963	tags=54%, list=29%, signal=39%	IER3/TNFRSF12A/IFITM3/BTG2/GADD45B/PPP2R5B/CTH/ISG20/SQSTM1/CD14/CASP4/AIFM3/SMAD7/DDIT3/CASP9/NEFH/RHOB/ATF3/GADD45A/BCL2L2/CLU/TSPO/NEDD9/JUN/GPX4/CCND1/RNASEL/IGF2R/CASP7/CAV1/GPX1/RARA/BAX/MMP2/DIABLO/BCL2L11/GPX3/IGFBP6/XIAP/FAS/DFFA/CASP3/F2R/FEZ1/PLAT/RELA/MCL1/ENO2/SAT1/TGFBR3/DAP3/CDKN1B/DNAJC3/TIMP1/APP/SC5D/RHOT2/PEA15/GSR/ROCK1/LMNA/TXNIP/GSN/HMOX1/ETF1/IFNGR1/PLPPR4/PSEN2/SPTAN1/PDGFRB/GSTM1

HALLMARK_INTERFERON_GAMMA_RESPONSE	127	0.411322	1.703154	0.001626	0.005659	0.002145	2535	tags=31%, list=19%, signal=26%	ISG15/PSMB8/IFITM2/BST2/C1S/IFITM3/CD40/UPP1/ISG20/CMKLR1/CASP4/HELZ2/MVP/HLA-A/PSMB10/IFI30/OGFR/LYSMD2/ST8SIA4/PELI1/ARID5B/VAMP5/PFKP/LY6E/CIITA/PSME2/CASP7/HLA-B/LAP3/IL2RB/RNF31/STAT3/IRF9/ST3GAL5/PSME1/NLRC5/FAS/CASP3/B2M/TAPBP
HALLMARK_INFLAMMATORY_RESPONSE	92	0.430141	1.70341	0.001695	0.005659	0.002145	2919	tags=37%, list=21%, signal=29%	BST2/TNFSF9/ABCA1/RGS16/MMP14/BTG2/CD40/CMKLR1/CD14/TNFRSF1B/OSMR/ITGB8/PTGER2/SPHK1/KLF6/BEST1/TPBG/SELENOS/SLC31A1/SLC11A2/LY6E/DCBLD2/IL2RB/PVR/PTGIR/ADORA2B/GABBR1/ATP2A2/INHBA/TAPBP/ADRM1/RELA/CD55/HBEGF
HALLMARK_ADIPOGENESIS	176	0.391482	1.717347	0.00156	0.005659	0.002145	2884	tags=32%, list=21%, signal=25%	HSPB8/SQOR/APOE/SPARCL1/ABCA1/DNAJB9/ESRRA/NKIRAS1/CD151/RNF11/TST/SLC27A1/FAH/BCL6/GADD45A/CHCHD10/LIPE/SLC1A5/SLC25A1/CMPK1/MGLL/GPX4/LIFR/ACO2/ALDH2/ENPP2/PIM3/TOB1/GRPEL1/PPP1R15B/RMDN3/MYLK/YWHAG/STOM/SCARB1/SORBS1/GPAT4/ADCY6/VEGFB/NABP1/ECHS1/SOWAHC/DHRS7/GPX3/G3BP2/PTGER3/PREB/MIGA2/AGPAT3/PFKFB3/ESYT1/IDH1/UQCR11/ALDOA/POR/PGM1
HALLMARK_MYOGENESIS	128	0.429621	1.774715	0.001637	0.005659	0.002145	2312	tags=32%, list=17%, signal=27%	HSPB8/ADAM12/KLF5/SYNGR2/KCNH2/SLC6A8/GADD45B/MYH8/PGAM2/KIFC3/COL6A3/PKIA/BIN1/PYGM/BDKRB2/COL6A2/SPHK1/CLU/PTGIS/CACNA1H/ATP6AP1/RIT1/COL4A2/AEBP1/SMTN/MAPRE3/SPARC/TCAP/SH3BGR/LSP1/BHLHE40/MYLK/ITGB5/NQO1/SORBS1/IGFBP7/TAGLN/SIRT2/TPM2/MYL6B/GPX3
HALLMARK_INTERFERON_ALPHA_RESPONSE	69	0.469101	1.78261	0.001698	0.005659	0.002145	3221	tags=36%, list=24%, signal=28%	ISG15/PSMB8/IFITM2/BST2/C1S/HLA-C/IFITM3/ISG20/HELZ2/IFI30/OGFR/LY6E/PSME2/LAP3/RNF31/IRF9/PSME1/PROCR/B2M/IRF2/IFIT3/NCOA7/TRIM25/LGALS3BP/GMPR
HALLMARK_KRAS_SIGNALING_UP	114	0.462171	1.884924	0.001675	0.005659	0.002145	1787	tags=29%, list=13%, signal=25%	PSMB8/PCSK1N/MMP9/SPARCL1/RGS16/YRDC/GADD45G/ALDH1A3/CTSS/CMKLR1/MAFB/CAB39L/TNFRSF1B/KIF5C/ENG/GLRX/SPRY2/FUCA1/NRP1/MYCN/ETV4/MMP11/IGF2/ABCB1/CROT/SCG5/KCNN4/DCBLD2/CXCR4/PPP1R15A/TMEM176B/LAT2/MAP3K1
HALLMARK_HYPOXIA	166	0.442195	1.915511	0.001582	0.005659	0.002145	1793	tags=30%, list=13%, signal=26%	TGFB3/IER3/STC1/MAFF/P4HA2/ZFP36/KDELR3/FOS/COL5A1/ISG20/GCK/HK2/PGAM2/IRS2/CHST3/PRDX5/GLRX/TES/DUSP1/DDIT3/STBD1/PYGM/CDKN1C/EFNA1/PGF/ATF3/TPST2/SLC6A6/KLF6/FBP1/EFNA3/RRAGD/SLC25A1/NCAN/NAGK/JMJD6/JUN/TPBG/PFKP/VEGFA/SLC2A3/DPYSL4/CXCR4/ALDOC/PPP1R15A/CA12/BHLHE40/MAP3K1/CAV1
HALLMARK_P53_PATHWAY	160	0.465649	2.002543	0.00159	0.005659	0.002145	2618	tags=38%, list=19%, signal=31%	OSGIN1/IER3/TNFSF9/SLC7A11/PLK2/RGS16/BTG2/FOS/UPP1/MKNK2/PLK3/TRIB3/EPHA2/DDIT3/IFI30/TSPYL2/DEF6/TRAF4/SLC3A2/ATF3/FUCA1/GADD45A/SPHK1/CTSD/CEBPA/AEN/MXD4/MAPKAPK3/BLCAP/CDK5R1/DCXR/JUN/H2AW/NINJ1/SEC61A1/FBXW7/RNF19B/DRAM1/PPP1R15A/TOB1/CCNG1/HRAS/WWP1/RPL18/SLC19A2/STOM/TRIAP1/JAG2/ABCC5/CCND3/BAX/RHBDF2/DDIT4/FAS/PROCR/COQ8A/HEXIM1/F2R/RPL36/BAK1/HINT1
HALLMARK_TNFA_SIGNALING_VIA_NFKB	140	0.533464	2.240042	0.001623	0.005659	0.002145	1652	tags=38%, list=12%, signal=34%	IER3/FOSL1/PHLDA2/MAFF/TNFSF9/ZFP36/ABCA1/PLK2/SIK1/YRDC/BTG2/SGK1/FOS/GADD45B/MSC/SQSTM1/IRS2/FJX1/PER1/CEBPB/DUSP1/MAP2K3/RELB/TNC/RHOB/EFNA1/ATF3/BCL6/EHD1/KLF9/GADD45A/SPHK1/DUSP4/KLF6/KDM6B/SERPINB8/JUNB/DNAJB4/HES1/FUT4/JUN/IER2/NR4A1/NINJ1/VEGFA/SLC2A3/CCND1/RNF19B/SPSB1/BCL3/DRAM1/SLC16A6/PPP1R15A
