# Supplementary material for: SAPCD2 promotes neuroblastoma progression by altering the subcellular distribution of E2F7
Source: Cell Death Dis. 2022 Feb 23;13(2):174. doi: 10.1038/s41419-022-04624-z (PMC8866461; doi:10.1038/s41419-022-04624-z)

Figure 1B

SH-SY5Y

SAPCD2

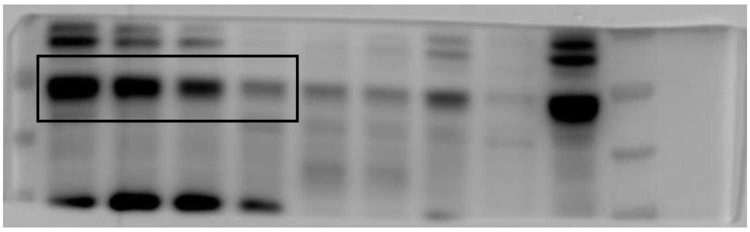

GAPDH

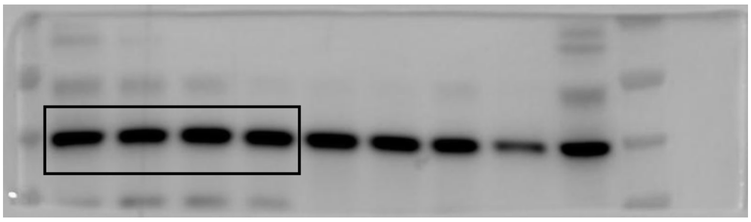

SK-N-BE(2)

SAPCD2

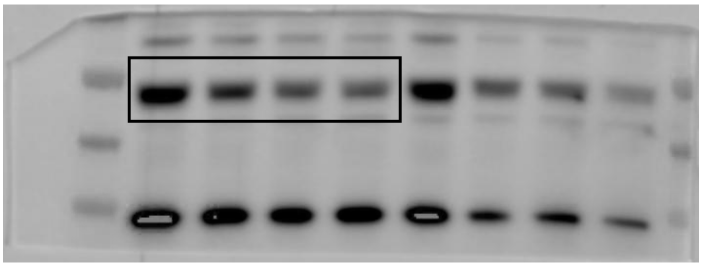

GAPDH

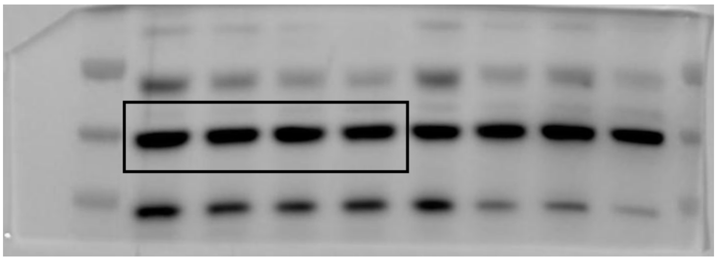

Figure 2A

SK-N-BE(2)

SAPCD2

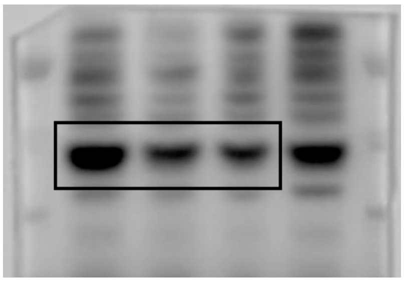

GAPDH

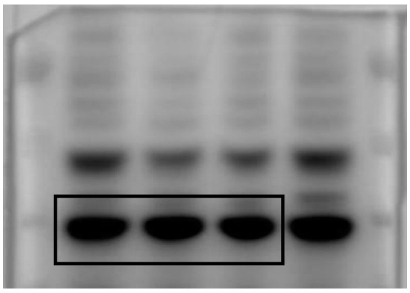

SH-SY5Y

SAPCD2

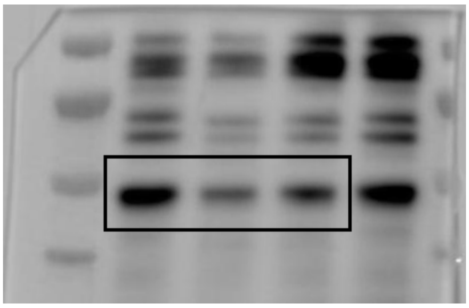

GAPDH

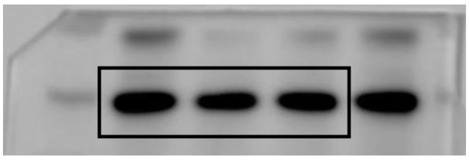

Figure 2G

SK-N-BE(2)

PARP

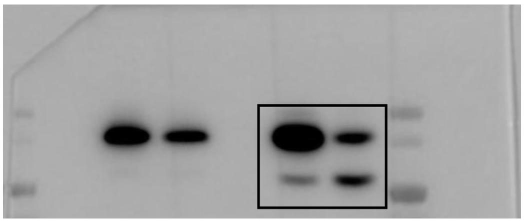

Caspase-3

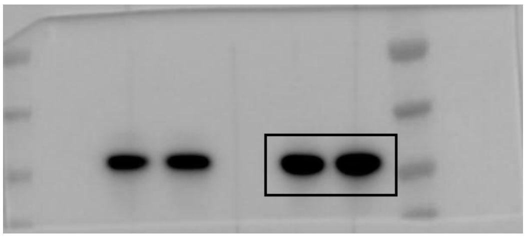

cleaved
caspase-3

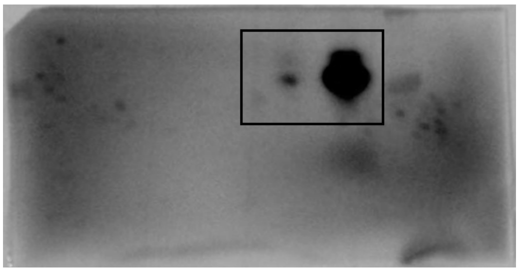

GAPDH

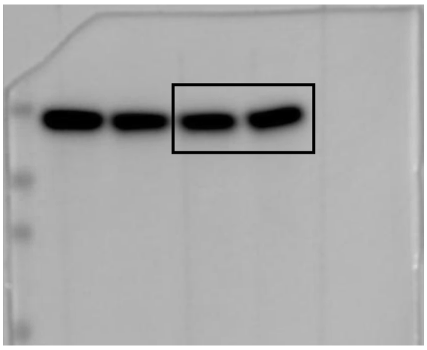

SH-SY5Y

PARP

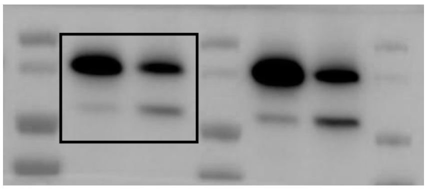

GAPDH

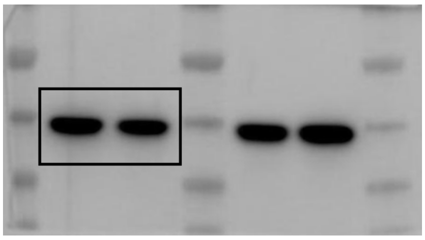

cleaved
caspase-3

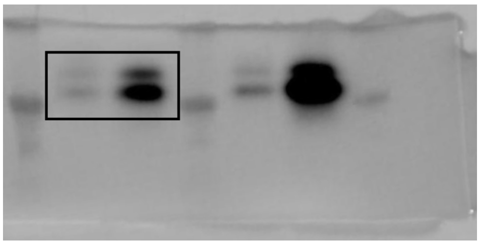

Caspase-3

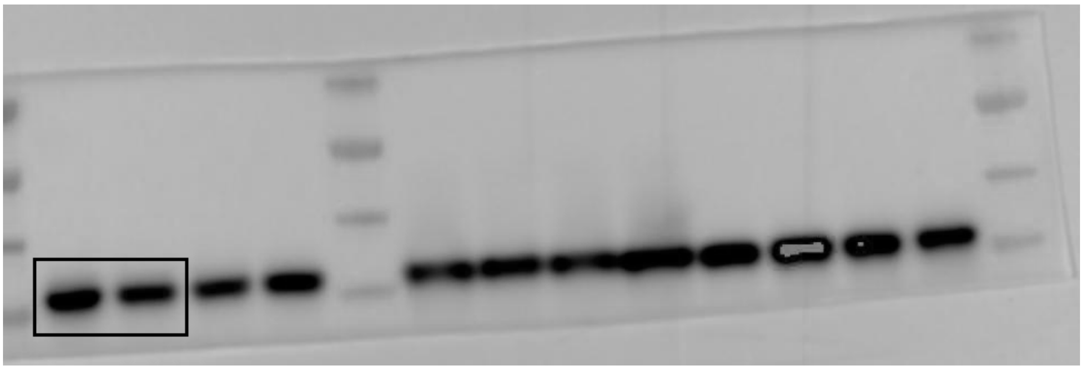

Figure 4B

E2F4

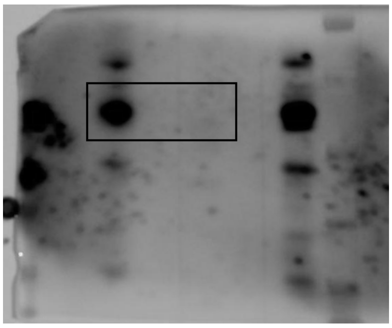

E2F7

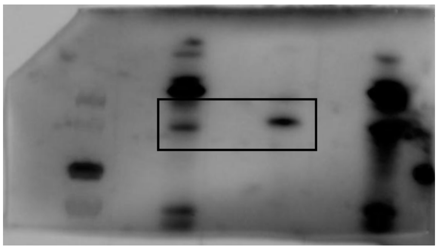

E2F1

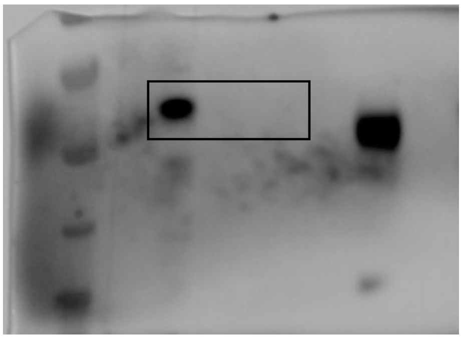

SAPCD2

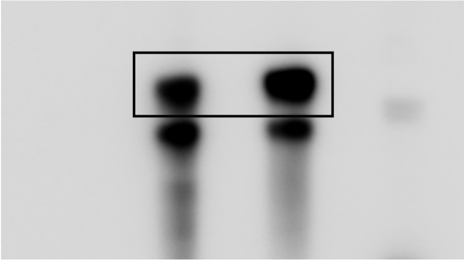

SAPCD2

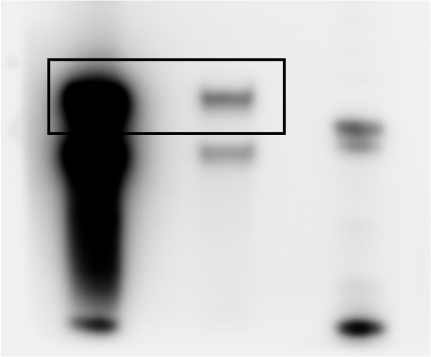

E2F7

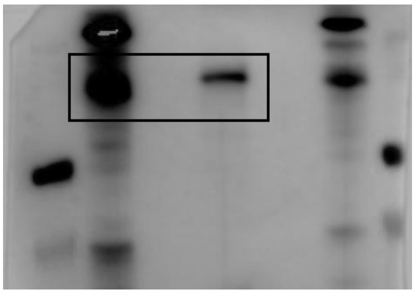

Figure 4E

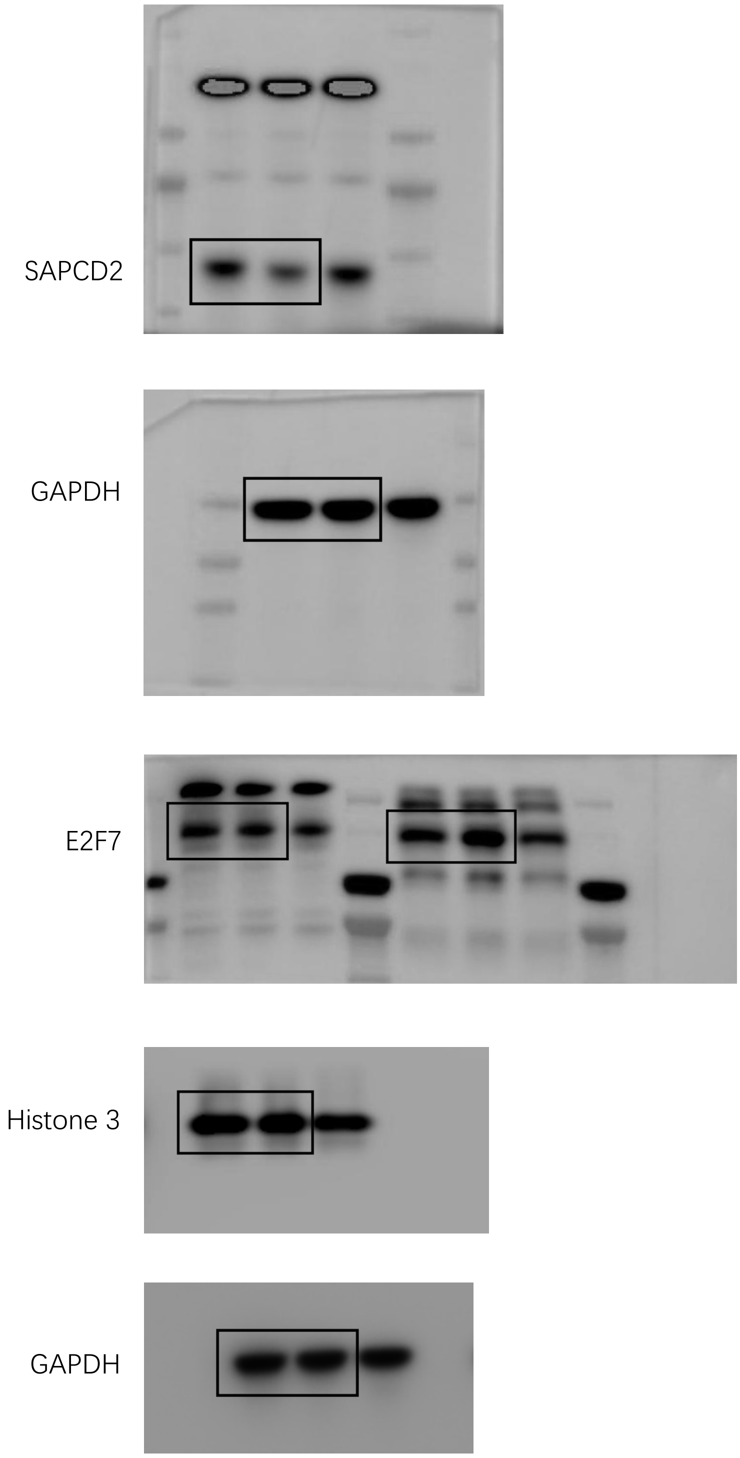

Supplement: Supplementary file 10 — Original Western Blots [file 41419_2022_4624_MOESM10_ESM.pdf]
