# Supplementary material for: SAPCD2 promotes neuroblastoma progression by altering the subcellular distribution of E2F7
Source: Cell Death Dis. 2022 Feb 23;13(2):174. doi: 10.1038/s41419-022-04624-z (PMC8866461; doi:10.1038/s41419-022-04624-z)
Supplement: Supplementary file 11 — Author Contribution Statement [file 41419_2022_4624_MOESM11_ESM.docx]

**AUTHOR CONTRIBUTIONS**

Z.M.Z. and H.B.C. conceived and performed most of the experiments; Z.H.L. and X.L.L. accomplished microarray study and some of the in vitro experiments; R.Z., X.M.L., Y.X., Y.L.C, J.J.Y, S.Q.J., R.D.Y., X.Y.G. and C.X.F. accomplished some of the in vitro experiments; R.Z., G.L. and D.W. accomplished some of the in vivo studies; Z.M.Z., F.F. and Y.Y. undertook the mining of publicly available datasets and performed the statistical analysis; Z.M.Z., Z.H.L., H.R.W., Y.F.T., Y.Y.X., G.H.Q. and J.P. secured funding; G.H.Q. and J.P. supervised the studies and critically revised the draft; Z.M.Z. and J.P. wrote the manuscript. All authors contributed to the article and approved the submitted version.
